# Exposure to sexually explicit media in early adolescence is related to risky sexual behavior in emerging adulthood

**DOI:** 10.1371/journal.pone.0230242

**Published:** 2020-04-10

**Authors:** Wen-Hsu Lin, Chia-Hua Liu, Chin-Chun Yi

**Affiliations:** 1 Institute of Health and Welfare Policy, School of Medicine, National Yang-Ming University, Taipei, Taiwan; 2 Department of Industrial Economics, College of Business and Management, Tamkang University, New Taipei City, Taiwan; 3 Institute of Sociology, Academia Sinica, Taipei, Taiwan; Spanish National Research Council, SPAIN

## Abstract

**Background:**

Sexually explicit media exposure during early adolescence has been found to be associated with risky sexual behavior. However, previous study suffered from methodological issue, such as selection bias. Furthermore, little is known about the effect of multi-modality sexually explicit media exposure on risky sexual behavior, and how this relationship can be applied to non-western societies.

**Objectives:**

This study aimed to improve upon previous studies by using instrumental variable estimation. In addition, this study also included multi-modality of sexually explicit media and three risky sexual behavior measure from a sample of Taiwanese adolescents.

**Methods:**

Participants were recruited from a prospective longitudinal study (Taiwan Youth Project). All were in 7^th^ grade (mean age = 13.3) when the study was initiated in 2000. Sexually explicit media exposure, including ever-exposure and number of modalities exposed to, was measured in wave 2 (8^th^ grade). Risky sexual behavior was measured in waves 8 (mean age = 20.3) and 10 (mean age = 24.3). A two-stage least squares regression was employed, with pubertal timing as the instrumental variable.

**Results:**

About 50% of participants had been exposed to sexual media content by 8^th^ grade, from an average of one modality. Sexually explicit media exposure predicted early sexual debut, unsafe sex, and multiple sexual partners (all: p < .05). Furthermore, exposure to more media modalities increased the likelihood of risky sexual behaviors. However, only the effect on early sexual debut was gender invariant.

**Conclusions:**

Exposure to sexually explicit media in early adolescence had a substantive relationship with risky sexual behavior in the emerging adulthood. Knowledge of this causal like effect provides a basis for building better preventive programs in early adolescence. One prominent way is early education on media literacy, and physicians themselves may need to be familiar with such content to initiate it.

## Introduction

Risky sexual behaviors, including early sexual debut, unsafe sex (e.g., inconsistent condom use), and multiple sexual partners (i.e., high partner change rate) [1], have received attention worldwide for their long-term negative impacts [2], particularly health-related, such as acquisition of sexually transmitted infections (STIs) [3], other diseases [4], unintended/teen pregnancy [3–5], and substance use [6]. Adolescents have received particular attention because they are among those most at risk of other STIs (e.g., gonorrhea) in many countries, such as the U.S. [7] and Taiwan [8] and for many parts of the world (e.g., Asia and Africa) they currently experiencing an HIV/AIDS epidemic [9]. Thus, there is a need to understand the early precursors to risky sexual behaviors for early prevention, as one of the best strategies to fight later negative outcomes.

Risky sexual behavior in adolescence is influenced by several important life domains, such as family/parents, peer, and individual factors. For example, several family-related factors, such as harsh parenting [10–11], low parental control [12], and family cohesion [13] have been identified as risk factors for sexual risk taking behavior and the underlying mechanisms are also presented (e.g., low parental control→low impulsive control→risky behavior or early maltreatment→negative emotions→risky behavior). Similarly, other studies argued from different theoretical perspectives and found possible precursors of risky sexual behaviors. For example, problem behavior theory [14] argues problem behaviors tend to cluster; hence, early substance use is highly related to later risky behavior, including risky sexual behaviors [15–16]. Similarly, social control theory [17] argued a lack of social bond (e.g., low school commitment) “releases” an individual for deviance, including risky sexual behaviors [18]. Other factors simply provide opportunities for sexual practice and are related to risky sexual behaviors, such as those in a romantic relationship [15, 19]. While these other factors have been related to risky sexual behaviors, studies have shown even controlling for these important precursors, one particular factor still has a strong relationship with risky sexual behaviors—sexual content in the media or sexually explicit media (SEM) [20–22]. Strasburger et al. [23] concluded sexual content in the media is a significant factor that influences children and teens in sexually-related behaviors, attitudes, and believes. Wright [24] mentioned exposure to SEM makes individuals more likely to change and establish promiscuous sexual attitudes, which are highly related to risky sexual behaviors later in life. Other studies demonstrated exposure to SEM is related to risky sexual behaviors because it changes viewer’s attitudes toward sexuality and women [25–26]. As such, one study argued, while the effects of sexual content in the media may be subtle, it is very important to control and measure [27]. Consequently, SEM may be essential when understanding risky sexual behaviors.

While exposure to SEM may make individual vulnerable to future risky sexual behavior, it is more so to adolescents for three reasons. First, SEM is not only prevalent, but is also influential during adolescence [28–30]. For example, Owens et al. [29] argued the proliferation of pornography has “influenced youth culture and adolescent development in unprecedented and diverse ways.” Second, adolescents are among the most frequent consumers of SEM [31–32] and perceive media depictions as real [32]. Furthermore, teenagers are affected by the way they interact (e.g., use and understand) the media and often allowing the media to influence and define their sex, love, and relationships [33]. Finally, in many developed countries, access to SEM is strongly and legally regulated, which makes it more attractive to youths because of the “forbidden fruit” effect [34].

The above reasoning suggests that adolescents and young adults are consumers of SEM and are susceptible to SEM. However, if the content of SEM is not “harmful,” exposure to SEM may not lead to negative consequences. For example, some have argued that SEM provides sexual education [35–36] and increases gender egalitarian attitudes [37]. Unfortunately, research has shown that the content of SEM overly depicts gratification of sexual behaviors and pays little or no attention to negative consequences [38], degrades women and “skew[s] away from intimacy and tenderness” (p.984) [39], and delivers an overly permissive sexual script [24]. Consequently, most previous studies have demonstrated that exposure to SEM during adolescence is related to early sexual debut [40–41], inconsistent condom use/unsafe sex [20, 25], and multiple sexual partners [42–43]. However, the “supposed” negative impact of SEM exposure and risky sexual behavior was not unequivocally found in other studies [44–48]. For example, a recent study found that SEM exposure was not related to either early sexual debut [48] or multiple sexual partners (i.e., greater than two sexual partners) [44].

Notwithstanding sampling variations and measurement differences, mixed results may also be due to omitted variable bias and/or self-selection bias (i.e., sexually active youths are more likely to view sexual content in the media) which prevent us from knowing the substantive relationship between SEM exposure and later risky sexual behavior [49–51]. As Tolman and McClelland argued [51], “the effects of seeing sexual media are plagued by a ‘chicken or egg’ challenge”; that is, whether youths who are sexually open are more likely to use SEM or adolescents become sexually active because of SEM exposure. Use of randomized controlled trials (RCTs), the “gold standard,” might also be inapplicable because of legal (e.g., presenting sexual content to minors) and ethical (e.g., assigning individuals to conditions that may compromise health) issues. Another common method to account for a self-selection bias is through a matching process. Three previous studies employed propensity score matching and all revealed that SEM exposure was not related to sexual initiation [46–47, 49]. However, propensity scores may be able to “eliminate” observable differences (i.e., matching on observable characteristics) but is limited in accounting for unobservable heterogeneity (i.e., unobservable differences). One means of rectifying these limitations is to use panel data to estimate the relationship, while including an instrumental variable (IV), as a means of approximating an RCT. Consequently, when properly used [52], the IV method provides a means of identifying a treatment effect from observational data (i.e., the substantive relationship).

Beside methodological limitations, whether exposure to various modalities of SEM will lead to a higher probability of risky sexual behavior has not received much research attention. Many previous studies have focused on only some types of sexually explicit material (e.g., X-rated movies or SEM websites) [44–48] and certain effects (e.g., early sexual debut or multiple sexual partners). To our knowledge, only one previous study examined the effect of exposure to several types of sexually explicit material and found that exposure to various SEM modalities was positively associated with the likelihood of casual sex and early sexual debut [31]. Given the mixed results of the relationship between SEM exposure and later risky sexual behavior and only one study that provided a more nuanced examination of the effects of multi-modal SEM exposure on risky sexual behavior, further study that accounts for methodological limitations and at the same time considers multi-modality SEM exposure and different risky sexual behaviors is warranted.

Finally, most previous studies have relied on Western samples (e.g., United States, United Kingdom, and European countries). SEM exposure and its relationship with risky sexual behaviors in somewhat more conservative societies (e.g., Asian countries) has been understudied. From the available current literature, it would appear that both SEM exposure and risky sexual behavior are quite different in Asian cultures than in Western countries. For example, studies from several East Asian countries showed that the SEM exposure rate among adolescents and young adults was around 50%: 4.5–57% in China [53], 40–43% in Taiwan [54] and Korea [55], and 9–53% in Hong Kong [56]; in contrast, studies from Western societies, including the United States [57], England [58], Sweden [59], Germany [60], and Australia [61] usually report exposure rates of 80% or higher. Similarly, using early onset of sexual behavior as an example, the proportion of adolescents who have sexual intercourse at young age (i.e., ≦16 or ≦ 14) is usually higher in Western society than in Asia [62–64]. Given these substantial differences, replicating the results from Western to a more conservative Eastern setting is important. Velezmoro and colleagues [65] have argued that studying sexual expression in different cultural settings sheds much light on the similarities and differences of the same phenomenon across cultures. Furthermore, some Asian countries suffer from increasing prevalence of STIs, such as an increased rate of HIV infection among young population in China [53, 66] and South Korea [67] and both HIV and other STIs (e.g., gonorrhea) are at their highest rates among adolescents and young adults (11–29) in Taiwan [8]. Although a few studies have been conducted and yielded similar results, these studies also suffered from the aforementioned limitations [68, 53–54].

## The present study

This study used IV estimation and a prospective cohort design to explore the relationship between SEM exposure in early adolescence and risky sexual behavior in emerging adulthood. We also examined the effects of multiple modalities of SEM (e.g., Internet and film) on risky sexual behavior. All the analyses were conducted using a sample from Taiwan, a more conservative society; hence, cross-cultural similarities and differences might be discovered [65]. We hypothesized that SEM exposure is related to later risky sexual behavior, and that the relationship would be stronger when adolescents used more SEM modalities. Finally, given that boys and girls experience physical development differently [69] and are socialized differently regarding sexual behavior [70], in addition to the main effect, we also stratified by gender to examine any differences in the relationship between SEM exposure and sexual behavior among males and females.

## Materials and methods

### Participants and study design

Data were drawn from the Taiwan Youth Project (TYP), a prospective cohort study of junior high school students from two cities (New Taipei City and Taipei) and one county (Yi-Lan County) in Northern Taiwan in that was initiated in 2000. In each selected school, two classes were randomly selected for each grade (7^th^ grade (J1) and 9^th^ grade (J3)), and all students in each selected class were recruited. Those who participated at baseline were followed annually until 2009 (wave 9), although some waves were not exactly one year apart. In 2011, the research team conducted wave 10, and have since completed two further follow-ups three years apart (wave 11 in 2014 and wave 12 in 2017). This study examined the J1 cohort (7^th^ grade) data from wave 1 (baseline; mean age = 13.3 (*SD* = .49)) to wave 10 (mean age = 24.3 (*SD* = .47)).

This study examined J1 cohort (7^th^ grade) data from wave 1 (baseline; mean age = 13.3 (SD = .49)) to wave 10 (mean age = 24.3 (SD = .47)). Approximately half the sample was male (51%). The sample size for examining early sexual debut and unsafe sex was 2,054, while that for multiple sexual partners was 1,477. The difference in sample size is due to different non-response rates. This drop of sample size occurred because the between-wave time lag was longer (i.e., two and half years between wave 9 and 10) compared to previous waves. The baseline data (wave 1) and wave 2 data (i.e., SEM exposure) were based on adolescents’ in-class self-report; in contrast, a parallel parental survey was used for parental education and family income, which conducted through in-home interview. For later waves of our subjects (wave 8, 9, and 10), in-home interview were conducted to collect all the data. At baseline (wave 1), all adolescents who agreed to participate gave oral consent. For these participated adolescents, one of their biological parents or legal guardians provided written consent. In addition, they were also invited to participate this research, and about 97% of them participated. The current study was approved by the internal review board at the National Yang Ming University (YM108005E) where the first author served as a faculty member.

## Measures

### Sexually explicit media exposure (wave 2)

This variable was measured at wave 2 (mean age = 14.3) using one question: “Have you ever seen any of the following adult-only or restricted (R-rated) media?” They were given a list of six media modalities: websites, magazines, comic books, novels, films, and other. While “adult-only” and “R-rated media” are not necessarily sexual in nature in many societies, the wording of the question in Mandarin (*Xian Zni Ji*) would be understood in Taiwanese society as referring to sexually explicit content (e.g., sexual intercourse and nudity). Hence, this item captured the intended SEM content. The items related to SEM exposure and sexual behavior were sensitive; hence, participants might be unwilling to report. To avoid this, all the TYP survey was self-report and was completed in the students’ classroom where only participating students and research team assistants were present. The research assistants explained to the students that no one other than the researchers would see the content of their survey and that all surveys were anonymous. Two variables were created to capture SEM exposure: multi-modality exposure and ever-exposure. For the former, we counted the number of modalities to which students were exposed, so the score ranged from 0 (no exposure) to 6 (used all six modalities). For the latter, participants were divided into SEM exposure (1) and non-exposure (0).

### Risky sexual behavior (wave 8-wave 10)

This variable included three behaviors: *early sexual debut*, *unsafe sex*, and *multiple sexual partners*. *Early sexual debut* was measured at wave 8 (mean age = 20.3). Each participant was asked to report his or her age of first sexual intercourse. Consensus on what age is considered to represent an early debut has not been reached in the literature, with various studies using different ages as the cut-off, such as 14 years old or younger [71], 16 years old or younger [72–73], or even 17/18 years old or younger [74]. Depending on the age used, the percentage of early initiators range from 17% [72] to 44% [73]. In the present study, 17 years of age or younger was used as the cut-off, which results in a percentage of about 11.9% (*n* = 245) of the sample being classified as early initiators. This cut-off is meaningful in the Taiwanese context for two reasons. First, age 18 is legally considered as adult. Furthermore, the summer of age 18 is the peak season during which adolescents lost their virginity because they graduated from high school and were about to enter college, which is also found in South Korea where educational system and culture is similar [75]. Second, the percentage of this cut-off is close to representative samples from high school students (10^th^-12^th^ grade), which showed that about 13% of high school students had had sexual intercourse already [76].

*Unsafe sex* was evaluated at wave 8 with a question on condom use during sexual intercourse (i.e., “Do you use condoms when you engage in sexual intercourse?”). The response categories included “no experience,” “always use a condom,” “sometimes use a condom,” and “do not use a condom most of the time.” Participants who selected the last two responses were considered to practice unsafe sex. Although this particular measure might be different from commonly used measures (e.g., condom use for recent sexual intercourse), it captured the respondents’ usual practice. Hence, it provided data regarding common condom use rather than just recent use or use in a particular situation. Hence, it captured the “true” meaning of unsafe sexual behavior. Based on this measure, the percentage of unsafe sexual practice is 18%.

Finally, at wave 10 (mean age = 24.3), participants were asked their lifetime number of sexual partners. This was used to gauge *multiple sexual partners*. The numbers ranged from 0 (no sexual experience) to 25 (mean = 1.76; SD = 2.46). Although the measure of risky sexual behavior may include various sexual behaviors, all of the behaviors assessed commonly increase individual’s risk of contracting STIs. As such, this study used early sexual debut, unsafe sex, and multiple sexual partners as three types of risky sexual behavior. One previous study used these three behaviors [1] and others used two of these three as the measure of risky sexual behavior [48]. Furthermore, early sexual debut and multiple sexual partners have been related to a high probability of unsafe sex and contraction of STIs [77–78]. While our measure may not exhaustive, it does include important risky sexual behaviors that have been assessed in previous studies.

### Pubertal timing (wave 1)

Pubertal timing was evaluated at wave 1 (mean age = 13.3) via self-report. For girls, four self-reported items from the Pubertal Development Scale (PDS) were employed [79]: pubic hair development, skin change, age of menarche, and growth spurt (α = .40). The response categories ranged from 1 (not yet begun) to 4 (fully developed). Girls were classified into three pubertal timing groups based on cut-offs of one standard deviation (*SD*) from the mean PDS score: (1) early (1 *SD* above the mean), (2) late (1 *SD* below the mean), and (3) on-time. For boys, we also used items from the PDS: change of voice, pubic hair development, beard development, skin change, and growth spurt (α = .68). The responses and grouping scheme were identical to those for girls. This grouping method has been used in previous studies [80–81] and the reliability and validity of the PDS have been confirmed [82]. The PDS has been shown to provide a suitable measure of puberty and to capture the subjective and social aspects of pubertal development [83]. However, while this measure has been validated in previous studies, it may not be able to capture a similar concept when used cross-culturally. Two indirect findings may address this concern. First, literature has shown that early pubertal timing is related to delinquency and depression [84–85], and two studies that used the same dataset as this study have demonstrated this relationship [80, 86]. Second, the distribution of age of menarche from a national representative sample of Taiwanese adolescents was very similar to the present sample (national representative sample: 82.8% before or at 7^th^ grade; current study: 88% before or at 7^th^ grade) [87]. In sum, the PDS provides a reasonable measure of pubertal development in Taiwan. In subsequent analyses, variation in PDS scores was used to create the IV.

### Control variables (wave 1 and wave 2)

The present study controlled for several potential confounders: gender [88], paternal education level, maternal education level [89], monthly family income [90], family intactness [91], number of siblings, presence of older siblings [92], parental control [93], family cohesion [94], academic performance [95], self-rated health [96], depressive symptoms [97], romantic relationship [98], and school fixed effect [99]. Each variable has been found to be related to either adolescent sexuality or SEM and risky sexual behavior. For example, family related variables (e.g., parental control and cohesion) captured a possibility that family and parents often play a central role in influencing adolescents’ deviant behaviors (i.e., SEM exposure and risky sexual behavior). Similarly, as mentioned above, problem social control may curtail adolescents’ unconventional behaviors, such as SEM use and risky sexual behavior. Furthermore, the social learning perspective may argue that sibling and peer effects play important roles in deviance during adolescence and emerging adulthood [100]; hence, we control for the number of sibling as well. Other factors (e.g., school) may create an environment where adolescents receive various exposures that may later influence their behaviors (e.g., sexual education). All variables were assessed at wave 1 or 2. Adolescent *gender* was coded as male (1) or female (0). Both *paternal* and *maternal education* levels were derived from the parental survey at wave 1 and were scored in three categories: lower than high school, high school, and junior college or above. In all subsequent analyses, two dummy variables were used with “lower than high school” as the reference group. *Monthly family income*, measured at wave 1 from the parental survey, was divided into five groups (based on new Taiwan dollars): less than 30,000, 30,000–50,000, 50,001–100,000, 100,001–150,000, and more than 150,000. Similarly, four dummy variables were used with “less than 30,000” as the reference category. *Family intactness* was a dichotomized variable with non-intactness as the reference group, which was based on wave 2 self-report. All the sibling measures were based on adolescents’ self-report at wave 1 and included the number of siblings each participant has and each siblings’ birth order. From this information, we created *number of siblings* and *presence of older siblings*. The latter included three groups: only child, yes, and no (reference group). *Parental control* was based on the summation of 5-dichotomized items that asked adolescents whether their parents control five daily activities (e.g., phone usage time and TV time). Higher scores indicated higher parental control. *Family cohesion* was based on the summation of six items that captured mutual family help and emotional attachment (e.g., “when I am down, I can receive comfort from my family”). Each item was based on 4-point Likert scale (i.e., “strongly disagree” to “strongly agree”). Higher scores indicated higher family cohesion. *Academic performance* was assessed with the question, “What is your class rank this semester?” The response categories were 1 (top 5), 2 (6–10), 3 (11–20), and 4 (over 21). *Health status* was based on self-rated health using five response categories. We grouped individuals into three categories: bad/very bad (reference group), fair, and good/very good. *Depressive symptoms* was a summation across a 7-item depressive symptom scale (e.g., “I feel depressed”), which was adopted from the Symptom Checklist-90-Revised (SCL-90-R) [101]. Each item was based on 5-point scale (i.e., no (0) to yes and very serious (4)). Summation across the seven items was used to calculate a total score. Dating experience was based on one item, which asked adolescents whether they have a boy/girlfriend. Finally, unobserved factors in school were controlled for by including *school fixed effect* in the subsequent analyses (descriptive statistics for all variables can be found in Table 1).

**Table 1:** Descriptive statistics for all variables.

	Full Sample^1^		Early sex debut/ Unsafe sex		Multiple sexual partner		Mean difference^3^ pubertal timing early/on-time vs. late	
	Mean	Std. Dev.	Mean	Std. Dev.	Mean	Std. Dev.	Coefficient	Std. Err.
**Dependent Variables**								
Early sex debut (first sex before age 17)			0.119	0.324			0.035**	(0.016)
Unsafe sex			0.181	0.385			0.039*	(0.021)
Number of sex partner (Min-Max: 0–25)					1.760	2.469	0.441***	(0.141)
**Control Variables**								
SEM exposure (wave 2)	0.502	0.500	0.504	0.500	0.509	0.500	0.091***	(0.023)
Multi-modality SEM: (Min-Max: 0–6)	1.018	1.367	1.019	1.366	1.007	1.324	0.228***	(0.070)
Comic books	0.324	0.468	0.327	0.469	0.336	0.472	0.060**	(0.026)
Novels	0.177	0.382	0.180	0.384	0.181	0.386	0.073***	(0.018)
Magazines	0.097	0.296	0.094	0.293	0.087	0.281	-0.004	(0.016)
Websites/webpage	0.185	0.389	0.185	0.389	0.177	0.382	0.054**	(0.026)
Films	0.227	0.419	0.226	0.418	0.219	0.414	0.037*	(0.019)
Others	0.007	0.086	0.007	0.085	0.007	0.082	0.009***	(0.002)
Pubertal timing: on-time (wave 1)^2^	0.687	0.464	0.692	0.462	0.693	0.462		
Pubertal timing: early	0.133	0.340	0.129	0.335	0.126	0.332		
Male (wave 1)	0.512	0.500	0.518	0.500	0.529	0.499	-0.197***	(0.035)
Father’s education: high school^2^	0.319	0.466	0.316	0.465	0.315	0.465	-0.006	(0.023)
Father’s education: junior college/above	0.240	0.427	0.235	0.424	0.238	0.426	-0.002	(0.024)
Mother’s education: below high school^2^	0.362	0.481	0.358	0.479	0.365	0.482	-0.014	(0.032)
Mother’s education: junior college/ above	0.149	0.356	0.146	0.353	0.146	0.353	-0.005	(0.020)
Monthly income: 30k-50k (NTD)^2^	0.268	0.443	0.274	0.446	0.279	0.449	-0.030	(0.022)
Monthly income: 50-100K	0.440	0.496	0.445	0.497	0.448	0.497	-0.033	(0.023)
Monthly income: 100K-150K	0.110	0.313	0.106	0.308	0.100	0.300	0.020	(0.015)
Monthly income: above 150K	0.035	0.183	0.032	0.176	0.028	0.166	-0.001	(0.012)
Family intactness (wave 2)	0.875	0.330	0.888	0.316	0.889	0.314	0.012	(0.023)
Number of sibling (wave 1)	2.622	0.896	2.628	0.873	2.642	0.856	-0.037	(0.061)
Only child (wave 1)	0.059	0.235	0.051	0.220	0.042	0.201	0.013	(0.013)
Presence of older sibling (wave 1)	0.569	0.495	0.564	0.496	0.564	0.496	-0.024	(0.030)
Parental control (wave 1)	2.630	1.659	2.579	1.642	2.588	1.633	-0.180*	(0.098)
Family cohesion (wave 1)	18.047	3.862	18.136	3.855	18.084	3.907	-0.295*	(0.173)
Class rank in 7th grade: ranked 6–10 (wave 1)^2^	0.182	0.386	0.192	0.394	0.208	0.406	0.015	(0.022)
Class rank in 7th grade: ranked 11–20	0.325	0.469	0.327	0.469	0.331	0.471	0.021	(0.031)
Class rank in 7th grade: ranked over 20	0.343	0.475	0.320	0.467	0.284	0.451	-0.024	(0.031)
Health status: fair (wave 2)^2^	0.299	0.458	0.293	0.455	0.289	0.453	0.015	(0.028)
Health status: good/very good	0.641	0.480	0.646	0.478	0.645	0.479	-0.024	(0.030)
Depressive symptom (wave 2)	3.364	3.671	3.287	3.618	3.350	3.657	0.379	(0.237)
Dating experience (wave 2)	0.129	0.336	0.114	0.318	0.114	0.318	0.046***	(0.014)
Joint test of covariates being zeros:^3^								
F statistics [p-value]							1.50	[0.149]
Observations	2,661		2,054		1,477			

^1^ The initial sample size in wave 1 (age 13.3) is 2690 but 29 individuals (1%) had missing value on the listed variables. SEM exposure measures are from wave 2 (age 14.3) with sample size 2, 568. Except for parental educational levels and monthly income, all variables are from either the wave 1 or wave 2 of student’ survey as indicated in the parentheses.

^2^ The reference groups for these variables are: “late” for *pubertal timing*; “below high school” for both *father’s education* and *mother’s education*; “below 30K” for *monthly income*; “ranked at 1–5” for *class rank in 7th grade*; “bad/very bad” for *health status*.

^3^ The 2,061 samples are used in computing the mean difference in each variable between early/on-time pubertal timing and late pubertal timing as well as the joint test of covariates being zeros. The joint test is conducted from the regression of *pubertal timing being early or on-time* on all control variables and junior high school fixed effects. This F-test examines whether the probability of pubertal timing being early or on-time is related to *class rank in 7th grade*, *health status*, *depressive symptom*, *father’s education*, *mother’s education*, *monthly income*, *intact family*, and *number of sibling*. Heteroskedasticity-robust standard errors in the parentheses clustered at the junior high school.

*** p<0.01,

** p<0.05,

* p<0.1.

### Statistical analysis

The linear probability model (LPM) based on the ordinary least squares (OLS) method was used to estimate the longitudinal effects of SEM exposure (ever-exposure and multi-modality exposure) during early adolescence on three risky sexual behaviors. While the convention for our outcomes may be using logit/probit model for dichotomized (i.e., early sexual debut and unsafe sex) and Poisson for count variable (i.e., multiple sexual partners), we employed OLS for several reasons. First, Hellevik [102] indicated that LPM is close to the logit model in most applications but has the advantage that its coefficients are easier to explain. Second, the main empirical model in the paper is two-stage least squares (2SLS) instrumental variable regressions, which is a linear model. Thus, regression analysis uses linear regression models or linear probability models for the convenience of comparison and intuition to convey the meaning of coefficients. While many covariates were controlled for, the estimated effect might still be biased due to unobserved confounding variables. Thus, to find a consistent, unbiased estimate of the effects of SEM exposure on risky sexual behaviors among adolescents, the 2SLS method with pubertal timing as the IV was used.

Variation in pubertal timing for the same cohort (*pubertal1*_*i*_ *and pubertal2*_*i*_) is used to instrument for SEM exposure (*y*_*sem*,*i*_) in the first stage, with controls of individual characteristics (*X*_*i*_) and junior high school fixed effects (*a*_*i*0_):

$$y_{sem,i}=a_{0i}+X_{i}^{’}\beta +\gamma_{1}{\left(pubertal1\right)}_{i}+\gamma_{2}{\left(pubertal2\right)}_{i}+v_{i}$$ (1)

where *y*_*sem*,*i*_ is the dependent for multi-modality SEM exposure and SEM exposure, respectively; the term *v*_*i*_ is the error term. The relationship between pubertal timing and SEM exposure should be positive. A *F* joint test is applied for testing the hypothesis that the coefficients on the instruments (i.e., pubertal timing) are all zero. When the corresponding *F*-statistic exceeds 10, then the instruments are strongly correlated with SEM exposure.

The second-stage equation estimate the effect of SEM exposure in early adolescence on risky sex behavior (*y*_*risky sexual behavior*_) in emerging adulthood:

$$y_{risky\;sexual\;behavior}=a_{0i}+X_{i}^{’}\beta +\gamma y_{sem,i}+u_{i}$$ (2)

where *y*_*risky sexual behavior*_ is risky sexual behavior for early sex debut, unsafe sex, and number of sex partners, respectively; individual characteristics (*X*_*i*_) and junior high school fixed effects (*a*_*i*0_) are the same as those in Eq (1) and the endogenous variable in (2) is the SEM exposure (*y*_*sem*,*i*_). We will separately estimate the effects of SEM-viewer and multi-modality SEM exposure on risky sexual behavior (all first stage analyses can be found in S1 Appendix).

Pubertal timing was set as the IV, as it fulfills the two main requirements of valid IVs: relevance and exogeneity [103]. The former requires the IV to be strongly related to the treatment (i.e., SEM exposure). Puberty is characterized by hormone elevation, and studies have shown that SEM exposure is prevalent during adolescence. Thus, individuals experiencing early puberty are more likely to be exposed to SEM than their counterparts, and this has been supported by numerous studies [104–105]. This requirement can also be statistically verified via the *F*-statistic (*F* > 10) in the first stage of a 2SLS [106]. Exogeneity, on the other hand, requires that the IV be uncorrelated with the error term in the regression equation. First, pubertal development is a biological process that almost all people experience. This development is influenced by genes and the environment, over which individuals have no control [107]. For example, twin studies have shown that approximately 50–80% of the variations in menarche timing are due to genetic factors and the remainder can be attributed to a non-shared environment or measurement error [108–109]. For the latter, as showed in the last column and the bottom of Table 1, the paper examines the possible correlation between pubertal timing and socioeconomic resources and did not find any significant correlation between pubertal timing and some observable socioeconomic resources (e.g., parental level of education and family monthly income). In addition, numerous environmental factors (e.g., school and family) were controlled for in the analyses, which might alleviate the concern of omitted variable bias. Accordingly, the IVs should be more likely to be uncorrelated with any of the unobserved factors that determined risky sexual behaviors. Furthermore, the estimated model included two IVs (two dummy variables). The over-identifying test (J-test) or the Sargan-Hansen test [110] can provide a statistical assessment of whether the estimated treatment effects are consistent in the 2SLS estimation.

While a valid IV design can provide causal estimates, attrition or missing data can still bias these estimates. This study used several methods to check for possible biases. First, our analytic sample was based on those who had information on SEM consumption in wave 2; the rate of missing data for all of the other explanatory variables including the instrumental variable (pubertal timing) was very low (See Table 1). Consequently, missing data on the right-hand side variable in the consequent analytic models may not be a serious issue. Second, the proportion of missing data on risky sexual behavior was not as low: 20% (514/2,568) for both early sexual debut and unsafe sex and 42% (1,091/2,568) for multiple sexual partners. Most of the missing data is due to attrition. For those who did not answer the first two risky sexual behavior questions (i.e., early sexual debut and inconsistent condom use), we imputed each item by checking their report on the same item at wave 9 or wave 10. However, for multiple sexual partners, we dropped those who did not provide a response. Third, we compared the distribution of the imputed sample to the original sample on pubertal timing, SEM exposure, and all of the control variables (see Table 1). As can be seen, the differences of the mean and *SD* between our various imputed samples and the original sample on all of the used variables were only minor. Finally, a Heckman selection model was used to see if attrition was related to risky sexual behavior. In this model, we used four variables as exclusion restrictions: housing type (e.g., live in a stand-alone house or an apartment), loving the current living area, neighborhood safety (e.g., “Do you think your neighborhood is safe?”), and number of years living at the current address. The results can be found in Table 2. From the bottom part of Table 2, one can find that the Wald tests indicated that the correlation between sample attrition and risky sexual behavior is not significant in all models (i.e., the two equations are independent of each other). In other words, attrition is not related to the decisions of engaging in risky sexual behaviors. These extra tests provided confidence that missing data on the outcome variables may be random. Consequently, the resulting estimates were unbiased but at the expense of the loss of precision and power because the standard errors were always larger than the estimates based on the full data. All statistical tests were based on 2-sided hypothesis tests with heteroskedasticity-robust standard errors adjusted for clustering at the junior high school level and were performed using Stata software (Stata 13.1; Stata Corp, College Station, TX).

**Table 2:** Selection models for relationship between non-missing and risky sex outcomes^1^.

	No missing	Early sex debut	No missing	Unsafe sex	No missing	Multiple sexual partner
Male (wave1)	0.035	0.052	0.037	0.085	0.152***	0.161**
	(0.082)	(0.073)	(0.083)	(0.068)	(0.055)	(0.063)
Multi-modality SEM (wave2)	0.034	0.151***	0.036	0.137***	0.012	0.174**
	(0.023)	(0.026)	(0.023)	(0.030)	(0.020)	(0.083)
Pubertal timing: on-time/ early (wave1)	0.015	0.172	0.010	0.128	-0.014	0.044
	(0.082)	(0.102)	(0.082)	(0.092)	(0.082)	(0.086)
Father’s education: high school^2^	-0.037	0.097	-0.036	-0.001	-0.007	0.120
	(0.072)	(0.107)	(0.070)	(0.088)	(0.069)	(0.108)
Father’s education: junior college/above	-0.089	0.225	-0.075	-0.031	-0.035	0.015
	(0.073)	(0.143)	(0.072)	(0.117)	(0.088)	(0.108)
Mom’s education: high school^2^	0.026	-0.063	0.033	-0.065	0.091	0.035
	(0.077)	(0.088)	(0.078)	(0.099)	(0.075)	(0.091)
Mom’s education: junior college/above	0.028	0.002	0.028	0.041	0.052	0.048
	(0.114)	(0.150)	(0.113)	(0.122)	(0.112)	(0.144)
Monthly income: 30K-50K (NTD)^2^	0.090	0.003	0.100	-0.031	-0.001	0.073
	(0.082)	(0.139)	(0.081)	(0.087)	(0.083)	(0.106)
Monthly income: 50K-100K	0.197	0.071	0.195	0.057	0.021	0.114
	(0.112)	(0.139)	(0.111)	(0.099)	(0.092)	(0.124)
Monthly income: 100K-150K	0.185	-0.070	0.175	0.009	-0.146	-0.077
	(0.136)	(0.192)	(0.136)	(0.135)	(0.109)	(0.166)
Monthly income: above 150K	0.034	0.169	0.024	0.200	-0.300	-0.032
	(0.247)	(0.235)	(0.241)	(0.211)	(0.176)	(0.215)
Intact Family (wave 2)	0.230***	-0.231**	0.235***	-0.216**	0.104	-0.210
	(0.087)	(0.104)	(0.085)	(0.098)	(0.085)	(0.238)
Number of sibling (wave1)	-0.026	0.015	-0.023	-0.055	0.014	-0.011
	(0.048)	(0.050)	(0.047)	(0.045)	(0.032)	(0.039)
Only child (wave1)	-0.293	-0.200	-0.285	-0.170	-0.396***	-0.206
	(0.154)	(0.209)	(0.157)	(0.207)	(0.127)	(0.222)
Presence of older sibling (wave1)	-0.103	-0.071	-0.106	0.049	-0.072	0.040
	(0.073)	(0.086)	(0.073)	(0.068)	(0.057)	(0.098)
Parental control (wave1)	-0.040	-0.002	-0.041**	-0.000	-0.012	0.013
	(0.022)	(0.021)	(0.021)	(0.018)	(0.016)	(0.030)
Family cohesion (wave 1)	0.001	-0.023**	0.001	-0.027***	-0.010	-0.012
	(0.008)	(0.010)	(0.008)	(0.009)	(0.007)	(0.008)
Class rank in 7th grade: 6–10 (wave1)^2^	-0.130	0.283**	-0.124	0.092	-0.098	0.030
	(0.097)	(0.120)	(0.101)	(0.116)	(0.090)	(0.099)
Class rank in 7th grade: 11–20	-0.259***	0.237	-0.257**	0.124	-0.324***	0.105
	(0.099)	(0.128)	(0.101)	(0.108)	(0.087)	(0.229)
Class rank in 7th grade: over 20	-0.422***	0.457***	-0.421***	0.275**	-0.621***	0.009
	(0.108)	(0.147)	(0.106)	(0.124)	(0.089)	(0.363)
Health status: fair (wave 2)^2^	-0.266**	0.074	-0.272**	0.251	-0.283**	0.067
	(0.120)	(0.214)	(0.121)	(0.212)	(0.126)	(0.236)
Health status: good/very good	-0.206	0.198	-0.208	0.420**	-0.223	0.160
	(0.135)	(0.223)	(0.136)	(0.192)	(0.116)	(0.214)
Depressive symptom (wave 2)	-0.016	0.007	-0.016**	0.006	-0.005	-0.003
	(0.008)	(0.014)	(0.008)	(0.010)	(0.007)	(0.010)
Dating experience (wave 2)	-0.367***	0.271**	-0.365***	0.412***	-0.193**	0.440
	(0.088)	(0.138)	(0.089)	(0.108)	(0.091)	(0.389)
Housing types: house^2^	0.102		0.108		0.054	
	(0.078)		(0.078)		(0.098)	
Housing types: other types	-0.110		-0.085		-0.109	
	(0.157)		(0.156)		(0.147)	
Love the current living area	-0.050		-0.058		-0.015	
	(0.089)		(0.087)		(0.122)	
Years of living	0.027***		0.026***		0.015***	
	(0.005)		(0.005)		(0.006)	
Years of living, squared	-0.000***		-0.000***		-0.000**	
	(0.000)		(0.000)		(0.000)	
Public security around home	0.065**		0.069**		-0.020	
	(0.031)		(0.033)		(0.029)	
Wald test for two independent equations:^1^						
Chi-squared statistic (p-value)	1.13	(0.287)	2.29	(0.130)	0.47	(0.491)
Observations	2,568	2,054	2,568	2,054	2,568	1477

^1^ The sample size for non-missing observations in *pubertal timing* and *SEM exposure* is 2, 568. The analytic samples for *early sex debut*, *unsafe sex* and *number of sex partner* models are 2054, 2054, and 1477. The *no missing* equals to 1 if the dependent variable is observed and 0 otherwise. Wald test with null hypothesis that the two equations are independent is applied. Except for parental educational levels, monthly income, housing types, years of living, and public security around home, all variables are from the waves 1 or 2 of student’ survey indicated in the parentheses.

^2^ The reference groups for these variables are: “below high school” for both *father’s education* and *mother’s education*; “below 30K” for *monthly income*;ranked at 1–5” for *class rank in 7th grade*; “bad/very bad” for *health status*; “Apartment” for *housing types*. All the models are estimated by using *housing types*, *years of living*, *squared years of living*, and *public security around home* as the exclusion restrictions. Heteroskedasticity-robust standard errors in the parentheses clustered at the junior high school.

*** p<0.01,

** p<0.05.

## Results

### Descriptive statistics

As indicated in Table 1, about half the adolescents (50%) were exposed to SEM in early adolescence, at an average of one modality (M = 1.02; SD = 1.37). The most common modality was comic books (32.7%) and the least common was magazines (9.4%). Overall, however, the prevalence of risky sexual behavior was low: early sexual debut, 11.9%; unsafe sex, 18.1%; average lifetime sexual partners was about 2. Gender differences were found in two out of three risky sexual behaviors (unsafe sex and number of sex partners), with males being more likely to be involved in these behaviors. In addition, a significant *t*-test result (*t* = -3.87; *p* < .01) indicated that males, on average, had more sexual partners (M = 1.99) than did females (M = 1.51). As can be seen, the most common SEM modality was comic books (32.7%), followed by films (22.7%). Surprisingly, only about 18.5% of adolescents used the Internet to view SEM. Additional analyses showed that more boys used each type of SEM more than girls did, with one exception: girls (22.5%) were more exposed to novels than were boys (13.7%). Furthermore, the *t*-test result (*t* = -7.2; *p* < .01) indicated that male adolescents, on average, used more types of SEM than female adolescents did.

### Sexually explicit media exposure and risky sexual behavior

**Fig 1:**
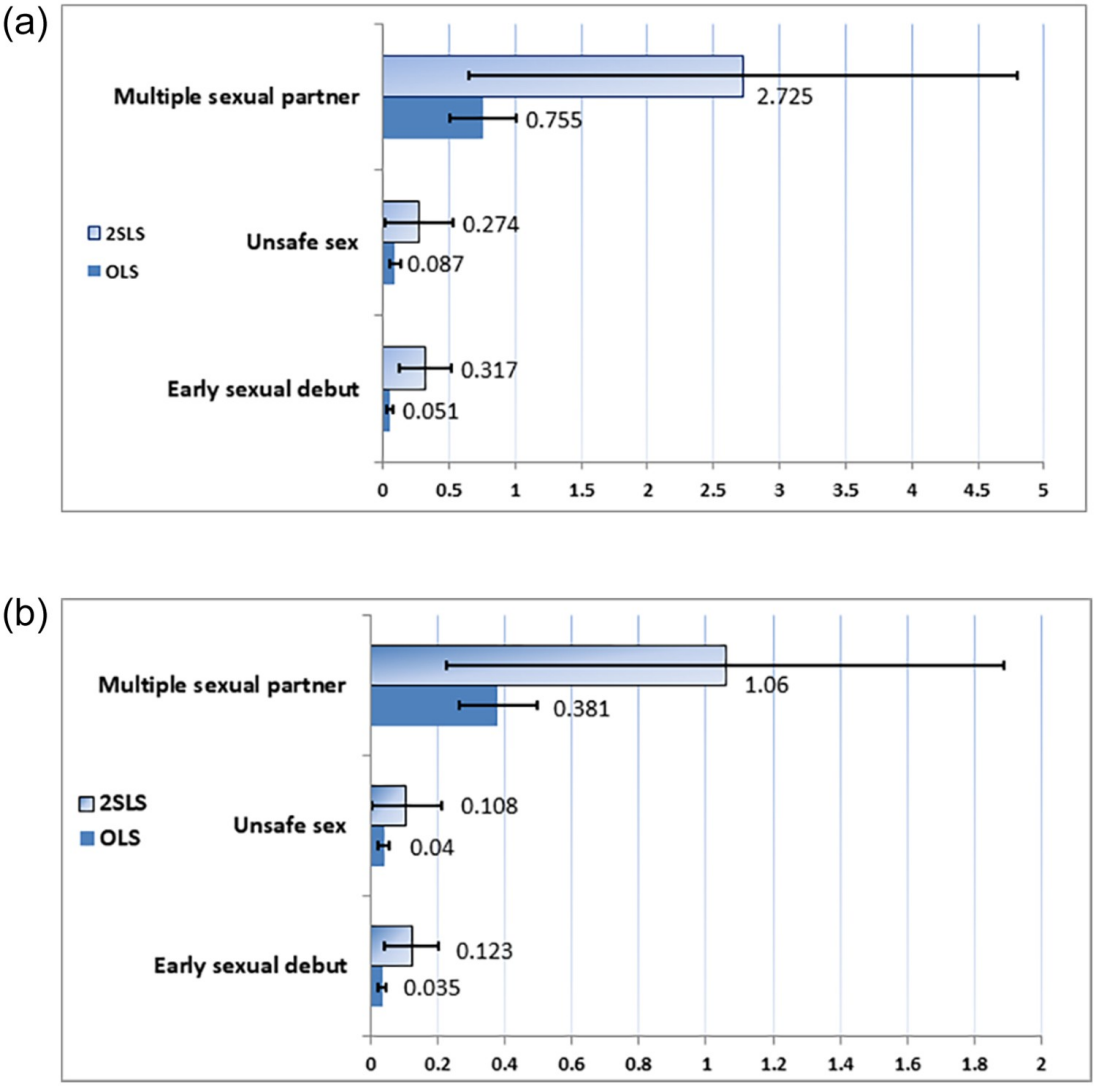
Main effects from OLS and 2SLS results. (a) The increased likelihood of early sexual debut and unsafe sex, and the increased number of sexual partner from SEM exposure for both OLS and 2SLS results (b) The increased likelihood of early sexual debut and unsafe sex, and the increased number of sexual partner for additional exposure to SEM for both OLS and 2SLS results.

A consistent finding (see Fig 1A and 1B) was that SEM exposure in early adolescence was significantly related to risky sexual behaviors in late adolescence (detail in S2 Appendix). Specifically, in Fig 1A and 1B, the results of the 2SLS estimation revealed that relative to their counterparts, adolescents exposed to SEM in early adolescence were 31.7% and 27.4% more likely to engage in sexual behavior before age 17 and to engage in unsafe sex, respectively. Moreover, these youths had on average three or more sexual partners by age 24. The estimated effects from the 2SLS models were 2.8 to 5.7 times larger than were those of the OLS estimations.

**Fig 2:**
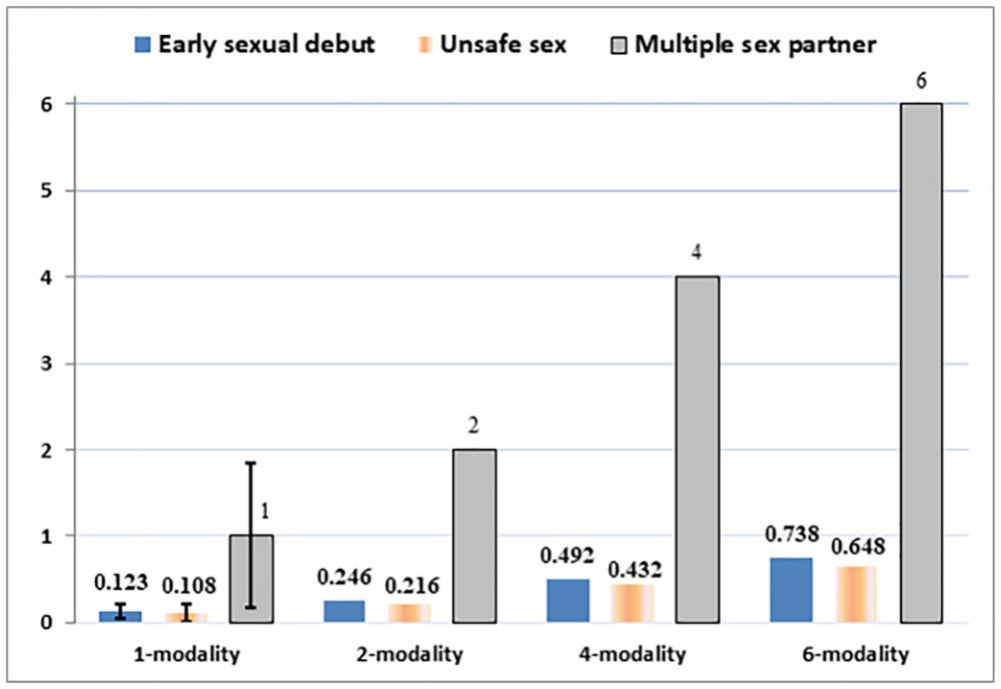
The effects of multi-modality exposure on the probability of risky sexual behavior and sexual partners.

As shown in Table 3, the effects of multi-modality SEM exposure on risky sexual behavior were also strong. The adolescents were 12.3% and 10.8% more likely to have made an early sexual debut and be involved in unsafe sex, respectively, when they viewed one or more SEM modalities during early adolescence compared to those who did not view any SEM. Of greater concern is that every modality during early adolescence led to, on average, one more sexual partner during late adolescence. The effect of multi-modality of SEM can be further understood by Fig 2 where we demonstrate the different probabilities of being involved in early sexual behavior and unsafe sex and the multiple sexual partners (to the nearest integer) at 1(mean), 2 (1 *SD*),4 (2 *SD*), and 6 (the highest) modalities. From the graphic, the trend clearly demonstrates that more exposure was related to a higher probability of risky sexual behavior and a greater number of sexual partners. The difference was pronounced between the mean (1 modality) and the extreme (6 modalities). The 2SLS estimations were 2.3 to 3.4 times larger than were those of the OLS. The results from above were consistent with those of previous studies that found SEM exposure is related to various risky sexual behaviors [20, 41–43, 56–57].

**Table 3:** Effects of multi-modality SEM exposure on risky sexual outcomes.

Mean of dependent variable	OLS Results			2SLS Results^1^		
	Early sex debut	Unsafe sex	Multiple sexual partner	Early sex debut	Unsafe sex	Multiple sexual partner
	0.119	0.181	1.76	0.119	0.181	1.76
	(1)	(2)	(3)	(4)	(5)	(6)
Multi-modality SEM (wave 2)	0.035***	0.040***	0.381***	0.123***	0.108**	1.060**
	(0.006)	(0.009)	(0.058)	(0.041)	(0.054)	(0.425)
Male (wave 1)	0.002	0.013	0.261**	-0.039	-0.019	-0.020
	(0.013)	(0.016)	(0.109)	(0.025)	(0.022)	(0.208)
Father’s education: high school^2^	0.016	-0.001	0.161	0.003	-0.011	0.017
	(0.021)	(0.022)	(0.147)	(0.022)	(0.026)	(0.206)
Father’s education: junior college/above	0.044	-0.004	0.039	0.040	-0.007	-0.003
	(0.028)	(0.029)	(0.221)	(0.027)	(0.029)	(0.214)
Mom’s education: high school^2^	-0.011	-0.013	0.101	-0.009	-0.011	0.102
	(0.018)	(0.027)	(0.159)	(0.019)	(0.027)	(0.160)
Mom’s education: junior college/above	0.002	0.015	0.060	0.007	0.020	0.094
	(0.030)	(0.029)	(0.281)	(0.032)	(0.028)	(0.275)
Monthly income: 30K-50K (NTD)^2^	-0.004	-0.014	-0.102	-0.007	-0.016	-0.194
	(0.027)	(0.023)	(0.180)	(0.027)	(0.023)	(0.187)
Monthly income: 50K-100K	0.006	0.001	0.069	-0.000	-0.004	-0.028
	(0.027)	(0.026)	(0.209)	(0.027)	(0.026)	(0.201)
Monthly income: 100K-150K	-0.020	-0.008	0.235	-0.035	-0.019	0.117
	(0.034)	(0.032)	(0.331)	(0.037)	(0.035)	(0.329)
Monthly income: above 150K	0.030	0.042	0.571	-0.020	0.004	0.234
	(0.052)	(0.060)	(0.509)	(0.064)	(0.075)	(0.599)
Family intactness (wave 2)	-0.061**	-0.070**	-0.488	-0.039	-0.053	-0.369
	(0.026)	(0.031)	(0.249)	(0.026)	(0.030)	(0.268)
Number of sibling (wave 1)	0.004	-0.012	-0.166**	0.003	-0.013	-0.147
	(0.009)	(0.011)	(0.069)	(0.009)	(0.011)	(0.080)
Only child (wave 1)	-0.026	-0.027	-0.399	-0.016	-0.019	-0.419
	(0.036)	(0.050)	(0.345)	(0.036)	(0.046)	(0.299)
Presence of older sibling (wave 1)	-0.009	0.015	-0.149	-0.014	0.011	-0.183
	(0.016)	(0.016)	(0.137)	(0.018)	(0.016)	(0.136)
Parental control (wave1)	0.001	0.002	0.071	-0.000	0.001	0.058
	(0.004)	(0.005)	(0.040)	(0.004)	(0.005)	(0.042)
Family cohesion (wave 1)	-0.005**	-0._007_***	-0.045**	-0.001	-0.004	-0.022
	(0.002)	(0.002)	(0.018)	(0.003)	(0.003)	(0.024)
Class rank in 7th grade: 6–10 (wave 1)^2^	0.047**	0.026	0.351**	0.043**	0.022	0.300
	(0.017)	(0.026)	(0.170)	(0.019)	(0.026)	(0.191)
Class rank in 7th grade: 11–20	0.041**	0.036	0.462***	0.042**	0.036	0.443**
	(0.019)	(0.024)	(0.156)	(0.019)	(0.025)	(0.174)
Class rank in 7th grade: over 20	0.094***	0.087***	0.631***	0.087***	0.081***	0.609***
	(0.025)	(0.028)	(0.164)	(0.027)	(0.028)	(0.172)
Health status: fair (wave 2)^2^	0.022	0.066	0.417	0.018	0.063	0.476
	(0.036)	(0.039)	(0.241)	(0.034)	(0.039)	(0.262)
Health status: good/very good	0.044	0.106***	0.626**	0.039	0.102***	0.651**
	(0.037)	(0.035)	(0.267)	(0.037)	(0.034)	(0.288)
Depressive symptom (wave 2)	0.002	0.002	0.020	-0.002	-0.001	-0.001
	(0.003)	(0.002)	(0.019)	(0.003)	(0.003)	(0.025)
Dating experience (wave 2)	0.092***	0.159***	1.287***	0.053	0.129***	1.075***
	(0.029)	(0.031)	(0.299)	(0.035)	(0.035)	(0.342)
School fixed effects	yes	yes	yes	yes	yes	Yes
First-stage F-statistic^3^				12.93	12.93	9.82
Overidentifying restrictions				0.012	0.285	1.772
J-statistic (p-value)				(0.914)	(0.594)	(0.1832)
Observations	2,054	2,054	1,477	2,054	2,054	1,477
R-squared	0.076	0.089	0.149			

^1^ The instrument variables (IVs) in two-stage least squares model (2SLS) are pubertal timing that included two dummy variables for students’ pubertal timing being on-time and being early, respectively. The multi-modality SEM exposure is the multiple type of SEM exposure (ranged from 0 to 6).

^2^ The reference groups for these variables are: “below high school” for both *father’s education* and *mother’s education*; “below 30K” for *monthly income*;“ranked at 1–5” for *class rank in 7th grade*; “bad/very bad” for *health status*. Heteroskedasticity-robust standard errors in the parentheses clustered at the junior high school.

*** p<0.01,

** p<0.05,

* p<0.1.

^3^ The first-stage F-statistic is the F-statistic testing the hypothesis that the coefficients on the IVs (i.e., *pubertal timing*) equal zero in first stage of 2SLS. Overidentifying test with null hypothesis that the two instruments are consistent with each other is applied.

Although SEM exposure was substantively related to later risky sexual behaviors, the estimated effects could be limited to a local average treatment effect (LATE) rather than an average treatment effect (ATE) [111], given that the estimated treatment effects would apply only to compliers (i.e., early maturers who also consumed SEM),and not to all participants, using the current statistical method. To address this issue, the models were estimated by enforcing a functional form so that the treatment effect might be applied to all participants (e.g., a bivariate Probit model for the ever-exposure variable with dichotomized outcomes). As shown in Table 4, the results indicated that all effects of SEM exposure on risky sexual behaviors remained significant, although the magnitudes were slightly reduced.

**Table 4:** Nonlinear structure estimations for effects of SEM on risky sexual outcomes^1^.

Mean of dependent variable	SEM exposure on risky sex outcomes			Multi-modality SEM on risky sex outcomes		
	Early sex debut	Unsafe sex	Multiple sexual partner	Early sex debut	Unsafe sex	Multiple sexual partner
	0.119	0.181	1.76	0.119	0.181	1.76
	(1)	(2)	(3)	(4)	(5)	(6)
**2SLS estimates:**^2^						
SEM exposure	0.317***	0.274**	2.725**			
	(0.100)	(0.131)	(1.059)			
Multi-modality SEM				0.123***	0.108**	1.060**
				(0.041)	(0.054)	(0.425)
**Nonlinear structure estimates:**^2^						
SEM exposure	0.318***	0.320***	2.447*			
	(0.086)	(0.074)	(1.267)			
Multi-modality SEM				0.109***	0.098**	0.950*
				(0.030)	(0.040)	(0.513)
Observations	2,054	2,054	1,477	2,054	2,054	1,477

^1^ Nonlinear structure estimations for (1) and (2), and for (4) and (5) are probit model with an endogenous binary explanatory variable, *SEM exposure*, and a continuous endogenous explanatory variable, *multi-modality SEM exposure*, respectively. The models (3) and (5) are poisson models with an endogenous explanatory variable for *SEM exposure* and *multi-modality SEM*, respectively. All the models are estimated by using two dummies for pubertal timing (i.e., the instrumental variables) as the exclusion restrictions.

^2^ The 2SLS estimates are reproduced for the ease of comparison. The control variables are the same as in 2SLS estimations. Heteroskedasticity-robust standard errors in the parentheses clustered at the junior high school.

*** p<0.01,

** p<0.05,

* p<0.1.

After confirming the main effect, this study further analyzed the effect by stratifying by gender. While the results remained the same in direction, the magnitude was lower for both gender groups. For boys, the results remained similar; that is, early exposure to SEM and the more modalities to which adolescent boys were exposed, the more likely they were to have first sexual intercourse early and more sexual partners. In contrast, the effects for females all decreased to nonsignificant levels except for early sexual debut. In other words, early exposure to SEM and exposure to more modalities of SEM increased the probability of early sexual intercourse for female adolescents in northern Taiwan. However, one must always keep in mind that all the effects were still in the right direction (i.e., positive effects). Given the reduced sample size, the decrease in magnitude was expected (See S3 Appendix).

## Discussion

Many studies have documented that early exposure to SEM may have various negative impacts on the development of risky sexual behavior. Risky sexual behavior has been linked to both physical (e.g., unwanted pregnancy and STIs) and mental (e.g., depression) problems. Furthermore, sexuality-related issues including sexual behavior and SEM exposure may vary across cultures; hence, understanding such relationships in more conservative cultures may provide further insights into this relationship. In addition, given the rise of STIs and teen pregnancy in many Asian countries [53, 66–67] and the call of WHO regarding global adolescent reproductive health [112], understanding the relationship could shed light on preventive strategies. These important considerations along with other limitations of previous studies (e.g., limited measurements of SEM and risky sexual behaviors and methodological limitations), indicated that further investigation of SEM exposure and risky sexual behavior was warranted. The purpose of this study was to build a stronger case for the relationship between SEM exposure and risky sexual behaviors, and at the same time to examine the effect of multi-modality of SEM exposure on three major risky sexual behaviors. Furthermore, this study also examined this relationship in a non-western society.

The results of this study were based on an IV estimation model which identified a causal like effect of SEM exposure on risky sexual behavior (at least for compliers). That is, early maturers who were exposed to SEM were also more likely to engage in risky sexual behavior. Our analyses consistently showed that early SEM exposure (8^th^ grade) is related to risky sexual behaviors in emerging adulthood including early sexual debut, unsafe sex, and multiple lifetime sexual partners. Although the unadjusted model (e.g., regular regression model) and the 2SLS regression both showed significant effects of early SEM exposure on later risky sexual behaviors, the magnitudes of all the estimated coefficients were stronger in the 2SLS models. Hence, the findings of this study not only echoed those of previous studies but also revealed that this relationship is substantive. These results could be understood from two theoretical perspectives. First, social learning theory [113] argues that behavior is learned via direct experience, vicarious experience from observing others (i.e., modeling), and complex cognitive operations (i.e., storing and processing information). Adolescents therefore “observe” behavior in SEM and learn how to perform it. They can also store and process information learned from the SEM (e.g., definitions or consequences of a behavior), thereby increasing or decreasing their likelihood of learning and applying related behavior. Similarly, Wright’s acquisition, activation, and application (AAA) model [114] explains that adolescents learn sexual scripts via this triple-A process: namely, they observe and acquire scripts from media, and from then on exposure to similar environmental cues will accentuate the learned scripts (“activation”). When the consequences of the scripted behavior are framed by media as more positive than negative, individuals are more likely to apply the script.

Besides general exposure (e.g., viewer vs. not), we further considered multi-modality of SEM use because Morgan [31] argued that such measure of SEM use is important. Our results showed that multi-modality of SEM use during early adolescence is also substantively related to risky sexual behaviors. In other words, the more modalities of SEM to which one is exposed, the higher probability of engaging in risky sexual behavior in emerging adulthood. The results are also consistent with both social learning theory [113] and the AAA [114] model because more exposure would accentuate the learned scripts and the favorable portrayal of similar behavior in the SEM. While in general dosage-effect is applied to the effect of frequency or intensity of exposure on behavior, some previous publications extend this relationship to accumulation negative experience of different kinds [115–116]. Specifically, Felitti [115] et al argued that their results was a dosage-effect because individuals who experience more different kinds of childhood adversities have lower level of health (e.g., low mental health).

Finally, provided that the functional forms assumed in the further analyses were correct, our results were very close to ATE, which in the present case is the difference in the mean of risky sexual behavior between treated (SEM exposure) and untreated (non-exposure) individuals within the entire population, not just an average treatment effect for a subpopulation (i.e., compliers). This gives us the confidence that early exposure to SEM may be detrimental to an individual’s reproductive health and such effects last into emerging adulthood.

Although our main effect was significant and strong, the effects were not omnibus when stratified by gender. While most effects were similar in terms of direction and magnitude, only early sexual debut and multiple sexual partners were significant for boys and early sexual debut for girls. These insignificant results could be due to a lack of power. The dramatic difference for girls might also be related to other important factors. For example, in a patriarchal society (e.g., China, Taiwan, and the U.S.), the gender double standard is very deep-rooted. Hence, while exposure to SEM might trigger early sexual intercourse three to four years later, the stigma for sexual promiscuity (i.e., multiple sexual partners) and lack of power to negotiate protection use may curtail the effects of SEM.

In sum, several strengths highlight our findings. First, our measures of SEM exposure and risky sexual behavior are broader than those used in many previous studies, which enabled this study to examine the relationship between multi-modality of SEM exposure and various risky sexual behaviors. This strength revealed an interesting dose-response-like relationship. Second, the dataset is a longitudinal prospective cohort dataset. This enabled us to employ instrumental variable estimation to account for the influence of unobserved factors and give proper time order. With this, this study revealed a substantive relationship between SEM exposure and risky sexual behavior. In addition, we checked the results by using models with more strict distributional assumptions (e.g., a bivariate probit model) and came to similar conclusions. Hence, we have some confidence that the estimated LATE is very close to the ATE. Furthermore, the analyses controlled for a variety of confounders such as health status, depressive symptoms and dating experience as well as school fixed effects to alleviate the influence of possible omitted variable bias. This gives us opportunities to examine similar results related to adolescent reproductive health in various cultures.

While the present results do provide invaluable insight into how sexually explicit media exposure affects later risky sexual behavior, some caveats must be addressed. First, the measurement of sexually explicit media exposure did not include frequency of exposure. Furthermore, the measure was static; hence, dynamic changes between sexually explicit media exposure and risky sexual behavior could not be explored [117]. Second, our measure of SEM included mostly non-Internet related media. This may provoke some concern when applying the results to the current era. To some extent, this may be a limitation to this study; however, given this study was conducted at the beginning of the surge in Internet usage, a limited measure of SEM exposure is understood. Although the Internet becomes the main media for entertainment and a main resource for SEM content, the influence of SEM from traditional media on risky sexual behaviors are continually found [20]. Hence, this limitation may not be a serious threat to the current study. However, discussion of three scenarios is worthwhile. First, given the vivid depiction of SEM on-line and becomes more “interactional,” our estimated effects of SEM from traditional media on risky sexual behaviors may be an underestimation of media effects. Second, Internet media usage may lead to reduced actual social contact, which may reduce sexual behaviors. For example, heavy Internet/problematic internet use may be related to lethargic negative emotions (i.e., loneliness and depression) [118], which can lead to lower levels of sexual activities. In this case, exposure to SEM on the Internet may reduce sexual behavior, in general, and risky sexual behavior, in particular; hence, our estimation may be overestimated. Third, one study has shown that dating applications (App) actually did not increase the possibility of building long-term romantic relationships, which may provide sexual opportunities. However, these Apps increased one kind of risky sexual behavior-casual sex (i.e., hook-up) [119]. In this final scenario, the effects of the Internet on risky sexual behaviors is positive but may be negative for general sexual behavior. While these are only some explanations and speculations, future studies should consider these issues.

Second, the requirement that the IV be uncorrelated with the second-stage error term can never be completely validated in empirical studies. The statistical analyses did show that the IV was reasonable, but this remains open to critique. For instance, although some studies have shown that pubertal timing is not related to later risky sexual behaviors [120–121], others have shown a partial relationship [122–123]. Hence, one may argue that there may be a direct link between pubertal timing and later risky sexual behavior. However, many previous studies did not consider the possible underlying mechanism linking pubertal timing and later risky sexual behavior (e.g., SEM exposure) and have indicated that the effects of early puberty on later behavior may be short-lived because all individuals eventually experience this change in young adulthood [122,124]. Given that we estimated the long-term effects of SEM exposure on risky sexual behaviors, we have some confidence in our IVs. Furthermore, the present results also demonstrated that the possible long-term effect of pubertal timing on risky sexual behavior is through SEM exposure (See Table 2 for the insignificant effect of pubertal timing on risky sexual behavior when controlling for SEM exposure). This result relieved the concern that pubertal timing has a direct and long-term effect on risky sexual behavior. Third, our outcome variable was limited to the three often used risky sexual behaviors; hence, our results may not be applicable to risky sexual behaviors other than these three risky sexual behavior. However, previous studies have shown that SEM exposure was significantly related to other risky sexual behavior or related outcomes, such as casual sex [31] and paid sex or group sex [125]. Fourth, all results were based on a self-report; consequently, reporting biases might have influenced the current results.

Medical and health researchers often argue that early prevention is a more efficient and better method of combating later diseases. Given the strong relationship between SEM exposure and risky sexual behaviors found in the present study, preventive strategies regarding SEM exposure should be implemented early in life, possibly before or at the start of puberty. This suggestion is corroborated by the American Academy of Pediatrics that indicated that early adolescence is the time to start sexuality discussions [126]. One possible preventive strategy is to cultivate adolescents’ media literacy, such as content literacy (i.e., knowledge about the ideas and contents presented in the media) and grammar literacy (i.e., knowledge of the techniques used to present visual content in the media, such as angles and zooms) [127]. To instill content literacy, officials (e.g., pediatricians and school teachers) and parents can take the initiative to provide adolescents with appropriate information on sexuality (e.g., ways of reducing sexual risk). To enhance grammar literacy, parents and school officials can help children decipher the scripts in SEM and “propagate” correct scripts (e.g., negative consequences of unsafe or casual sex). One recent review showed that a media literacy intervention was effective in preventing the negative impact of media on risky adolescent behavior [127]. In addition, sex education implementing positive information, such as preventive (e.g., risk avoidance) and protective behaviors (e.g., STIs protection), may have a great impact on adolescent sexual health. In fact, one study showed that receiving correct information enhanced individuals’ protective actions against future risky behaviors [128]. However, given the sensitive nature of these topics, before school officials and parents aim to cultivate adolescents’ media literacy or provide sex-related information, confidentiality between the two parties must be established [129]. Finally, aside from our main finding, our first-stage of 2SLS results showed that family cohesion is related to a lower probability of SEM exposure; hence, encouraging parents to establish a warm and mutually supportive family atmosphere may help to reduce SEM exposure, which in turn can help in reducing future sexual risk taking.

## Conclusion

Two important results emerged from this study. First, sexually explicit media exposure in early adolescence was strongly related to three risky sexual behaviors—early sexual debut, unsafe sex, and sexual partners—in late adolescence, and this relationship was very close to causal. Second, the association was dose-response, such that using more modalities of sexually explicit media led to a higher probability of being involved in risky sexual behavior later in life. Given that the negative consequences of risky sexual behaviors (e.g., STIs and unplanned pregnancy) have tremendous social costs in both Western and Asian societies, it is necessary to implement preventive strategies early on.

## Supporting information

S1 Appendix (DOCX)

S2 Appendix (DOCX)

S3 Appendix (DOCX)

## Abbreviations

ATE: Average Treatment Effect
HIV/AIDS: human immunodeficiency virus/acquired immunodeficiency syndrome
IV: Instrumental Variable
OLS: Ordinary Least Squares
PDS: Pubertal Development Scale
RCT: Randomize Control Trial
SLS: Two Stage Least Squares
STIs: sexually transmitted infections
TYP: Taiwan Youth Project
