# Supplementary material for: Exposure to sexually explicit media in early adolescence is related to risky sexual behavior in emerging adulthood
Source: PLoS One. 2020 Apr 10;15(4):e0230242. doi: 10.1371/journal.pone.0230242 (PMC7147756; doi:10.1371/journal.pone.0230242)
Supplement: S1 Appendix — (DOCX) [file pone.0230242.s001.docx]

Table A First Stage Results for Determinants of SEM exposure

|  | Binary SEM^1^ | |  | Multi-modality SEM^1^ | |
| --- | --- | --- | --- | --- | --- |
| Mean of dependent variable | 0.504 | 0.509 |  | 1.02 | 1.01 |
|  | (1) | (2) |  | (3) | (4) |
| Pubertal timing: on-time (wave 1)^2^ | 0.104*** | 0.102*** |  | 0.285*** | 0.273*** |
|  | (0.025) | (0.030) |  | (0.071) | (0.083) |
| Pubertal timing: early | 0.207*** | 0.209*** |  | 0.530*** | 0.541*** |
|  | (0.035) | (0.046) |  | (0.116) | (0.146) |
| Male (wave 1) | 0.113*** | 0.100*** |  | 0.481*** | 0.414*** |
|  | (0.029) | (0.036) |  | (0.083) | (0.096) |
| Father's education: high school^2^ | 0.030 | 0.027 |  | 0.151 | 0.204 |
|  | (0.028) | (0.034) |  | (0.090) | (0.101) |
| Father's education: junior college or above | -0.021 | -0.038 |  | 0.048 | 0.056 |
|  | (0.036) | (0.046) |  | (0.092) | (0.113) |
| Mom's education: high school^2^ | -0.009 | 0.006 |  | -0.015 | 0.007 |
|  | (0.034) | (0.040) |  | (0.079) | (0.093) |
| Mom's education: junior college or above | -0.014 | 0.020 |  | -0.053 | -0.030 |
|  | (0.046) | (0.056) |  | (0.149) | (0.180) |
| Monthly income: 30K-50K (NTD)^2^ | 0.051 | 0.081 |  | 0.061 | 0.140 |
|  | (0.030) | (0.044) |  | (0.072) | (0.096) |
| Monthly income: 50K-100K | 0.068 | 0.084 |  | 0.095 | 0.152 |
|  | (0.037) | (0.047) |  | (0.097) | (0.102) |
| Monthly income: 100K-150K | 0.092 | 0.100 |  | 0.175 | 0.163 |
|  | (0.061) | (0.072) |  | (0.143) | (0.177) |
| Monthly income: above 150K | 0.213*** | 0.166** |  | 0.572*** | 0.509** |
|  | (0.063) | (0.080) |  | (0.190) | (0.211) |
| Family intactness (wave 2) | -0.116*** | -0.061* |  | -0.269*** | -0.174* |
|  | (0.031) | (0.038) |  | (0.083) | (0.101) |
| Number of sibling (wave 1) | 0.008 | -0.008 |  | 0.014 | -0.024 |
|  | (0.016) | (0.019) |  | (0.038) | (0.048) |
| Only child (wave 1) | -0.046 | -0.009 |  | -0.111 | 0.033 |
|  | (0.057) | (0.058) |  | (0.160) | (0.207) |
| Presence of older sibling (wave 1) | 0.020 | 0.009 |  | 0.060 | 0.055 |
|  | (0.025) | (0.029) |  | (0.061) | (0.077) |
| Parental control (wave1) | 0.010 | 0.013 |  | 0.018 | 0.022 |
|  | (0.007) | (0.008) |  | (0.019) | (0.022) |
| Family cohesion (wave 1) | -0.011*** | -0.011*** |  | -0.034*** | -0.033*** |
|  | (0.003) | (0.003) |  | (0.009) | (0.010) |
| Class rank in 7th grade: 6-10 (wave 1)^2^ | 0.011 | 0.023 |  | 0.056 | 0.089 |
|  | (0.031) | (0.039) |  | (0.078) | (0.110) |
| Class rank in 7th grade: 11-20 | -0.024 | 0.009 |  | -0.001 | 0.039 |
|  | (0.027) | (0.031) |  | (0.069) | (0.092) |
| Class rank in 7th grade: over 20 | -0.005 | 0.011 |  | 0.094 | 0.056 |
|  | (0.031) | (0.035) |  | (0.079) | (0.093) |
| Health status: fair (wave 2)^2^ | 0.014 | -0.039 |  | 0.049 | -0.081 |
|  | (0.050) | (0.060) |  | (0.161) | (0.187) |
| Health status: good/very good | 0.027 | -0.011 |  | 0.066 | -0.031 |
|  | (0.059) | (0.072) |  | (0.166) | (0.191) |
| Depressive symptom (wave 2) | 0.012*** | 0.012** |  | 0.042*** | 0.030*** |
|  | (0.004) | (0.005) |  | (0.011) | (0.010) |
| Dating experience (wave 2) | 0.120*** | 0.102** |  | 0.406*** | 0.264** |
|  | (0.034) | (0.045) |  | (0.089) | (0.106) |
| School fixed effects | yes | yes |  | yes | yes |
| Observations | 2,054 | 1,477 |  | 2,054 | 1,477 |
| R-squared | 0.114 | 0.112 |  | 0.141 | 0.126 |

**Notes**: ^1^ The sample sizes are 2,054 and 1,477 for *early sex debut* and *unsafe sex* models and for *number of sex partner* model, respectively. Except for parental educational levels and monthly income, all variables are from either the wave 1 or wave 2 of student’ survey as indicated in the parentheses.

^2^ The reference groups for these variables are: “late” for *pubertal timing*; “below high school” for both *father's education* and *mother's education*; “below 30K” for *monthly income;* “ranked at 1-5” for *class rank in 7th grade*; “bad/very bad” for *health status*;. Heteroskedasticity-robust standard errors in the parentheses clustered at the junior high school. *** p<0.01, ** p<0.05, * p<0.1.

Table B First Stage Results for Determinants of SEM exposure, by Gender

|  | Male Sample^1^ | | | | |  | Female Sample^1^ | | | | |
| --- | --- | --- | --- | --- | --- | --- | --- | --- | --- | --- | --- |
|  | Binary SEM exposure | |  | Multi-modality SEM | |  | Binary SEM | |  | Multi-modality SEM | |
| Mean of dependent variable | 0.547 | 0.548 |  | 1.22 | 1.18 |  | 0.458 | 0.466 |  | 0.807 | 0.815 |
|  | (1) | (2) |  | (3) | (4) |  | (1) | (2) |  | (3) | (4) |
|  |  |  |  |  |  |  |  |  |  |  |  |
| Pubertal timing: on-time (wave 1)^2^ | 0.118*** | 0.132*** |  | 0.361*** | 0.407*** |  | 0.078 | 0.046 |  | 0.163* | 0.079 |
|  | (0.036) | (0.040) |  | (0.107) | (0.127) |  | (0.049) | (0.058) |  | (0.081) | (0.117) |
| Pubertal timing: early | 0.194*** | 0.185*** |  | 0.562*** | 0.549*** |  | 0.169** | 0.211** |  | 0.324** | 0.471** |
|  | (0.048) | (0.060) |  | (0.169) | (0.199) |  | (0.073) | (0.087) |  | (0.150) | (0.217) |
| Father's education: high school^2^ | 0.053 | 0.045 |  | 0.144 | 0.213 |  | 0.022 | 0.005 |  | 0.213** | 0.207** |
|  | (0.038) | (0.046) |  | (0.140) | (0.161) |  | (0.034) | (0.041) |  | (0.088) | (0.095) |
| Father's education: junior college/above | -0.045 | -0.069 |  | -0.040 | 0.020 |  | -0.034 | -0.085 |  | 0.046 | -0.058 |
|  | (0.050) | (0.068) |  | (0.162) | (0.185) |  | (0.060) | (0.077) |  | (0.131) | (0.168) |
| Mom's education: high school^2^ | -0.062 | -0.005 |  | -0.144 | -0.019 |  | 0.037 | 0.027 |  | 0.088 | 0.053 |
|  | (0.039) | (0.053) |  | (0.117) | (0.152) |  | (0.049) | (0.055) |  | (0.087) | (0.095) |
| Mom's education: junior college/above | -0.035 | 0.055 |  | -0.163 | -0.072 |  | 0.012 | 0.019 |  | 0.135 | 0.133 |
|  | (0.058) | (0.081) |  | (0.222) | (0.279) |  | (0.076) | (0.088) |  | (0.167) | (0.213) |
| Monthly income: 30K-50K (NTD)^2^ | 0.042 | 0.041 |  | 0.020 | -0.028 |  | 0.078** | 0.121** |  | 0.130 | 0.253** |
|  | (0.046) | (0.063) |  | (0.140) | (0.189) |  | (0.037) | (0.051) |  | (0.083) | (0.114) |
| Monthly income: 50K-100K | 0.083 | 0.062 |  | 0.145 | 0.058 |  | 0.061 | 0.117** |  | 0.027 | 0.180 |
|  | (0.053) | (0.060) |  | (0.168) | (0.192) |  | (0.046) | (0.057) |  | (0.116) | (0.116) |
| Monthly income: 100K-150K | 0.098 | 0.097 |  | 0.206 | 0.121 |  | 0.118 | 0.140 |  | 0.183 | 0.209 |
|  | (0.082) | (0.087) |  | (0.244) | (0.290) |  | (0.090) | (0.116) |  | (0.174) | (0.194) |
| Monthly income: above 150K | 0.281*** | 0.184 |  | 0.704** | 0.494 |  | 0.133 | 0.178* |  | 0.323 | 0.419* |
|  | (0.087) | (0.117) |  | (0.289) | (0.353) |  | (0.093) | (0.099) |  | (0.290) | (0.234) |
| Family intactness (wave 2) | -0.061 | -0.015 |  | -0.192 | -0.125 |  | -0.175*** | -0.115* |  | -0.322** | -0.197 |
|  | (0.042) | (0.058) |  | (0.141) | (0.178) |  | (0.051) | (0.065) |  | (0.122) | (0.156) |
| Number of sibling (wave1) | 0.011 | 0.003 |  | 0.011 | -0.023 |  | 0.004 | -0.014 |  | -0.006 | -0.034 |
|  | (0.024) | (0.028) |  | (0.063) | (0.071) |  | (0.019) | (0.024) |  | (0.041) | (0.053) |
| Only child (wave1) | -0.057 | -0.041 |  | -0.097 | -0.048 |  | -0.051 | 0.045 |  | -0.140 | 0.080 |
|  | (0.070) | (0.077) |  | (0.209) | (0.246) |  | (0.077) | (0.107) |  | (0.169) | (0.276) |
| Presence of older sibling (wave1) | 0.062 | 0.040 |  | 0.133 | 0.093 |  | -0.022 | -0.023 |  | 0.009 | 0.018 |
|  | (0.037) | (0.046) |  | (0.092) | (0.128) |  | (0.033) | (0.034) |  | (0.080) | (0.075) |
| Parental control (wave1) | 0.014 | 0.015 |  | 0.029 | 0.038 |  | 0.004 | 0.011 |  | 0.003 | 0.004 |
|  | (0.010) | (0.013) |  | (0.031) | (0.036) |  | (0.010) | (0.014) |  | (0.024) | (0.035) |
| Family cohesion (wave 1) | -0.010** | -0.008 |  | -0.035** | -0.022 |  | -0.013*** | -0.013*** |  | -0.040*** | -0.044*** |
|  | (0.004) | (0.006) |  | (0.014) | (0.017) |  | (0.004) | (0.004) |  | (0.011) | (0.013) |
| Class rank in 7th grade: 6-10 (wave 1)^2^ | 0.056 | 0.122* |  | 0.174 | 0.340* |  | -0.021 | -0.071 |  | -0.054 | -0.129 |
|  | (0.053) | (0.062) |  | (0.133) | (0.180) |  | (0.060) | (0.071) |  | (0.127) | (0.161) |
| Class rank in 7th grade: 11-20 | -0.016 | 0.048 |  | 0.122 | 0.233 |  | -0.024 | -0.029 |  | -0.104 | -0.147 |
|  | (0.051) | (0.055) |  | (0.129) | (0.177) |  | (0.057) | (0.060) |  | (0.120) | (0.125) |
| Class rank in 7th grade: over 20 | 0.013 | 0.078 |  | 0.247** | 0.338* |  | -0.020 | -0.047 |  | -0.056 | -0.191 |
|  | (0.049) | (0.056) |  | (0.120) | (0.179) |  | (0.052) | (0.054) |  | (0.112) | (0.137) |
| Health status: fair (wave 2)^2^ | 0.035 | -0.063 |  | 0.271 | -0.104 |  | -0.002 | -0.017 |  | -0.076 | -0.071 |
|  | (0.073) | (0.085) |  | (0.271) | (0.328) |  | (0.068) | (0.080) |  | (0.186) | (0.186) |
| Health status: good/very good | 0.041 | -0.044 |  | 0.201 | -0.124 |  | 0.024 | 0.026 |  | 0.032 | 0.072 |
|  | (0.086) | (0.101) |  | (0.269) | (0.316) |  | (0.077) | (0.097) |  | (0.188) | (0.201) |
| Depressive symptom (wave 2) | 0.013*** | 0.014** |  | 0.043*** | 0.027** |  | 0.011** | 0.010 |  | 0.039*** | 0.031** |
|  | (0.005) | (0.006) |  | (0.014) | (0.012) |  | (0.006) | (0.006) |  | (0.014) | (0.013) |
| Dating experience (wave 2) | 0.138*** | 0.145** |  | 0.543*** | 0.310** |  | 0.117** | 0.085 |  | 0.244* | 0.234 |
|  | (0.050) | (0.061) |  | (0.121) | (0.127) |  | (0.052) | (0.058) |  | (0.129) | (0.144) |
| Constant | 0.547*** | 0.676*** |  | 0.843 | 0.814 |  | 0.772*** | 0.706*** |  | 1.529*** | 1.353*** |
|  | (0.182) | (0.208) |  | (0.514) | (0.666) |  | (0.120) | (0.153) |  | (0.356) | (0.361) |
| School fixed effects | yes | yes |  | yes | yes |  | yes | yes |  | yes | yes |
| Observations | 1,064 | 781 |  | 1,064 | 781 |  | 990 | 696 |  | 990 | 696 |
| R-squared | 0.141 | 0.147 |  | 0.155 | 0.146 |  | 0.140 | 0.168 |  | 0.177 | 0.198 |

**Notes**: ¹ For male sample, the sample sizes are 1,064 and 781 for *early sex debut* and *unsafe sex* models and for *number of sex partner* model, respectively. While female sample, the sizes are 990 and 696 for *early sex debut* and *unsafe sex* models and for *number of sex partner* model, respectively.

² The reference groups for these variables are: “late” for *pubertal timing*; “below high school” for both *father's education* and *mother's education*; “below 30K” for *monthly income;* “ranked at 1-5” for *class rank in 7th grade*; “bad/very bad” for *health status*; Heteroskedasticity-robust standard errors in the parentheses clustered at the junior high school. *** p<0.01, ** p<0.05, * p<0.1.
