# Supplementary material for: Exposure to sexually explicit media in early adolescence is related to risky sexual behavior in emerging adulthood
Source: PLoS One. 2020 Apr 10;15(4):e0230242. doi: 10.1371/journal.pone.0230242 (PMC7147756; doi:10.1371/journal.pone.0230242)
Supplement: S2 Appendix — (DOCX) [file pone.0230242.s002.docx]

Table A Effects of SEM exposure on Risky Sexual Outcomes

|  | OLS Results | | |  | 2SLS Results^1^ | | |
| --- | --- | --- | --- | --- | --- | --- | --- |
|  | Early | Unsafe | Number of |  | Early | Unsafe | Number of |
|  | sex debut | sex | sex partner |  | sex debut | sex | sex partner |
| Mean of dependent variable | 0.119 | 0.181 | 1.76 |  | 0.119 | 0.181 | 1.76 |
|  | (1) | (2) | (3) |  | (4) | (5) | (6) |
|  |  |  |  |  |  |  |  |
| SEM exposure (wave 2) | 0.051*** | 0.087*** | 0.755*** |  | 0.317*** | 0.274** | 2.725** |
|  | (0.014) | (0.020) | (0.126) |  | (0.100) | (0.131) | (1.059) |
| Male (wave1) | 0.013 | 0.023 | 0.342*** |  | -0.017 | 0.002 | 0.144 |
|  | (0.014) | (0.017) | (0.111) |  | (0.020) | (0.016) | (0.158) |
| Father's education: high school^2^ | 0.020 | 0.003 | 0.219 |  | 0.012 | -0.003 | 0.159 |
|  | (0.021) | (0.022) | (0.146) |  | (0.021) | (0.023) | (0.170) |
| Father's education: junior college/above | 0.047 | -0.000 | 0.089 |  | 0.052 | 0.004 | 0.158 |
|  | (0.028) | (0.028) | (0.235) |  | (0.027) | (0.028) | (0.255) |
| Mom's education: high school^2^ | -0.011 | -0.013 | 0.098 |  | -0.008 | -0.010 | 0.093 |
|  | (0.018) | (0.026) | (0.166) |  | (0.019) | (0.026) | (0.179) |
| Mom's education: junior college/above | 0.001 | 0.014 | 0.031 |  | 0.005 | 0.018 | 0.007 |
|  | (0.029) | (0.029) | (0.298) |  | (0.028) | (0.028) | (0.310) |
| Monthly income: 30K-50K (NTD)^2^ | -0.005 | -0.016 | -0.110 |  | -0.016 | -0.024 | -0.267 |
|  | (0.027) | (0.022) | (0.184) |  | (0.029) | (0.022) | (0.211) |
| Monthly income: 50K-100K | 0.005 | -0.002 | 0.063 |  | -0.010 | -0.013 | -0.096 |
|  | (0.027) | (0.025) | (0.214) |  | (0.027) | (0.027) | (0.227) |
| Monthly income: 100K-150K | -0.019 | -0.009 | 0.223 |  | -0.042 | -0.026 | 0.017 |
|  | (0.034) | (0.032) | (0.332) |  | (0.038) | (0.035) | (0.347) |
| Monthly income: above 150K | 0.039 | 0.046 | 0.638 |  | -0.017 | 0.007 | 0.320 |
|  | (0.050) | (0.059) | (0.523) |  | (0.059) | (0.069) | (0.608) |
| Family intactness (wave 2) | -0.064** | -0.071** | -0.508 |  | -0.035 | -0.050 | -0.386 |
|  | (0.026) | (0.031) | (0.252) |  | (0.027) | (0.031) | (0.273) |
| Number of sibling (wave1) | 0.004 | -0.012 | -0.170** |  | 0.002 | -0.014 | -0.151 |
|  | (0.009) | (0.011) | (0.070) |  | (0.010) | (0.012) | (0.084) |
| Only child (wave1) | -0.027 | -0.027 | -0.381 |  | -0.015 | -0.019 | -0.361 |
|  | (0.036) | (0.052) | (0.374) |  | (0.038) | (0.050) | (0.358) |
| Presence of older sibling (wave1) | -0.008 | 0.015 | -0.136 |  | -0.013 | 0.012 | -0.149 |
|  | (0.016) | (0.016) | (0.137) |  | (0.018) | (0.017) | (0.139) |
| Parental control (wave1) | 0.001 | 0.002 | 0.069 |  | -0.001 | -0.000 | 0.047 |
|  | (0.004) | (0.005) | (0.040) |  | (0.004) | (0.005) | (0.041) |
| Family cohesion (wave 1) | -0.005** | -0.007*** | -0.049** |  | -0.002 | -0.005 | -0.026 |
|  | (0.002) | (0.002) | (0.018) |  | (0.003) | (0.003) | (0.023) |
| Class rank in 7th grade: 6-10 (wave1)^2^ | 0.048*** | 0.027 | 0.366** |  | 0.046** | 0.026 | 0.331 |
|  | (0.017) | (0.026) | (0.171) |  | (0.019) | (0.026) | (0.182) |
| Class rank in 7th grade: 11-20 | 0.042** | 0.038 | 0.469*** |  | 0.049*** | 0.043 | 0.461*** |
|  | (0.019) | (0.024) | (0.154) |  | (0.017) | (0.024) | (0.163) |
| Class rank in 7th grade: over 20 | 0.097*** | 0.091*** | 0.642*** |  | 0.100*** | 0.093*** | 0.639*** |
|  | (0.026) | (0.028) | (0.168) |  | (0.026) | (0.029) | (0.177) |
| Health status: fair (wave 2)^2^ | 0.023 | 0.067 | 0.415 |  | 0.019 | 0.064 | 0.495 |
|  | (0.037) | (0.040) | (0.252) |  | (0.036) | (0.040) | (0.280) |
| Health status: good/very good | 0.045 | 0.106*** | 0.622** |  | 0.038 | 0.101*** | 0.648** |
|  | (0.038) | (0.036) | (0.272) |  | (0.039) | (0.036) | (0.297) |
| Depressive symptom (wave 2) | 0.003 | 0.003 | 0.023 |  | -0.001 | 0.001 | -0.001 |
|  | (0.003) | (0.003) | (0.019) |  | (0.003) | (0.003) | (0.027) |
| Dating experience (wave 2) | 0.101*** | 0.165*** | 1.315*** |  | 0.064** | 0.140*** | 1.078*** |
|  | (0.029) | (0.030) | (0.295) |  | (0.033) | (0.031) | (0.339) |
| School fixed effects | yes | yes | yes |  | yes | yes | yes |
| First-stage F-statistic^3^ |  |  |  |  | 14.03 | 14.03 | 10.00 |
| Overidentifying restrictions |  |  |  |  | 0.001 | 0.486 | 1.893 |
| J-statistic (p-value) |  |  |  |  | (0.970) | (0.486) | (0.169) |
| Observations | 2,054 | 2,054 | 1,477 |  | 2,054 | 2,054 | 1,477 |
| R-squared | 0.063 | 0.083 | 0.133 |  |  |  |  |

**Notes**: ¹The instrument variables (IVs) in two-stage least squares model (2SLS) are pubertal timing that included two dummy variables for students’ pubertal timing being on-time and being early, respectively. The SEM exposure is a binary variable indicating that students ever exposed to SEM.

² The reference groups for these variables are: “below high school” for both *father's education* and *mother's education*; “below 30K” for *monthly income;* “ranked at 1-5” for *class rank in 7th grade*; “bad/very bad” for *health status*; Heteroskedasticity-robust standard errors in the parentheses clustered at the junior high school. *** p<0.01, ** p<0.05, * p<0.1.

^3^ The first-stage F-statistic is the F-statistic testing the hypothesis that the coefficients on the IVs (i.e., *pubertal timing*) equal zero in first stage of 2SLS. Overidentifying test with null hypothesis that the two instruments are consistent with each other is applied.
