# Supplementary material for: Exposure to sexually explicit media in early adolescence is related to risky sexual behavior in emerging adulthood
Source: PLoS One. 2020 Apr 10;15(4):e0230242. doi: 10.1371/journal.pone.0230242 (PMC7147756; doi:10.1371/journal.pone.0230242)
Supplement: S3 Appendix — (DOCX) [file pone.0230242.s003.docx]

Table A Effects of SEM exposure on Risky Sexual Outcomes for Male

|  | OLS Results | | |  | 2SLS Results^1^ | | |
| --- | --- | --- | --- | --- | --- | --- | --- |
|  | Early | Unsafe | Number of |  | Early | Unsafe | Number of |
|  | sex debut | sex | sex partner |  | sex debut | sex | sex partner |
| Mean of dependent variable | 0.130 | 0.201 | 1.985 |  | 0.130 | 0.201 | 1.985 |
|  | (1) | (2) | (3) |  | (4) | (5) | (6) |
|  |  |  |  |  |  |  |  |
| SEM exposure (wave 2) | 0.043** | 0.085*** | 0.859*** |  | 0.286** | 0.196 | 2.260* |
|  | (0.019) | (0.030) | (0.182) |  | (0.133) | (0.190) | (1.280) |
| Father's education: high school^2^ | -0.001 | -0.021 | 0.161 |  | -0.015 | -0.027 | 0.089 |
|  | (0.026) | (0.034) | (0.233) |  | (0.029) | (0.033) | (0.243) |
| Father's education: junior college/above | 0.058 | 0.010 | 0.064 |  | 0.068* | 0.015 | 0.154 |
|  | (0.040) | (0.048) | (0.351) |  | (0.039) | (0.048) | (0.356) |
| Mom's education: high school^2^ | -0.005 | -0.014 | 0.051 |  | 0.012 | -0.006 | 0.068 |
|  | (0.029) | (0.040) | (0.264) |  | (0.031) | (0.040) | (0.252) |
| Mom's education: junior college/above | -0.010 | -0.030 | 0.085 |  | -0.001 | -0.025 | 0.021 |
|  | (0.048) | (0.037) | (0.372) |  | (0.048) | (0.037) | (0.376) |
| Monthly income: 30K-50K (NTD)^2^ | -0.014 | -0.053 | -0.335 |  | -0.020 | -0.056 | -0.385 |
|  | (0.045) | (0.040) | (0.262) |  | (0.042) | (0.038) | (0.281) |
| Monthly income: 50K-100K | -0.006 | -0.020 | -0.318 |  | -0.023 | -0.028 | -0.391 |
|  | (0.043) | (0.041) | (0.310) |  | (0.040) | (0.043) | (0.314) |
| Monthly income: 100K-150K | -0.004 | -0.030 | 0.211 |  | -0.025 | -0.039 | 0.089 |
|  | (0.048) | (0.059) | (0.629) |  | (0.047) | (0.062) | (0.597) |
| Monthly income: above 150K | 0.029 | 0.045 | 0.629 |  | -0.037 | 0.014 | 0.381 |
|  | (0.074) | (0.087) | (0.878) |  | (0.073) | (0.112) | (0.953) |
| Intact Family (wave 2) | 0.010 | 0.004 | -0.019 |  | 0.021 | 0.009 | -0.003 |
|  | (0.035) | (0.038) | (0.299) |  | (0.035) | (0.038) | (0.320) |
| Number of sibling (wave 1) | 0.007 | -0.022 | -0.251** |  | 0.004 | -0.023 | -0.253** |
|  | (0.018) | (0.017) | (0.120) |  | (0.018) | (0.017) | (0.121) |
| Only child (wave 1) | -0.012 | -0.015 | -0.441 |  | 0.004 | -0.008 | -0.373 |
|  | (0.048) | (0.073) | (0.499) |  | (0.047) | (0.071) | (0.483) |
| Presence of older sibling (wave 1) | 0.006 | 0.013 | -0.081 |  | -0.008 | 0.006 | -0.129 |
|  | (0.030) | (0.028) | (0.203) |  | (0.032) | (0.028) | (0.209) |
| Parental control (wave1) | -0.001 | -0.002 | 0.102* |  | -0.004 | -0.003 | 0.085 |
|  | (0.006) | (0.006) | (0.058) |  | (0.006) | (0.007) | (0.061) |
| Family cohesion (wave 1) | -0.002 | -0.005 | -0.031 |  | 0.000 | -0.004 | -0.020 |
|  | (0.003) | (0.004) | (0.027) |  | (0.004) | (0.004) | (0.028) |
| Class rank in 7th grade: 6-10 (wave 1)^2^ | 0.069* | 0.066 | 0.544* |  | 0.056 | 0.060 | 0.384 |
|  | (0.036) | (0.040) | (0.287) |  | (0.038) | (0.040) | (0.333) |
| Class rank in 7th grade: 11-20 | 0.069** | 0.085** | 0.535** |  | 0.072** | 0.087*** | 0.472* |
|  | (0.031) | (0.033) | (0.254) |  | (0.031) | (0.032) | (0.271) |
| Class rank in 7th grade: over 20 | 0.137*** | 0.144*** | 0.800*** |  | 0.135*** | 0.144*** | 0.711** |
|  | (0.039) | (0.041) | (0.265) |  | (0.040) | (0.039) | (0.284) |
| Health status: fair (wave 2)^2^ | 0.056 | 0.127*** | 0.485 |  | 0.050 | 0.124*** | 0.590 |
|  | (0.050) | (0.044) | (0.395) |  | (0.050) | (0.042) | (0.407) |
| Health status: good/very good | 0.075 | 0.178*** | 0.826** |  | 0.067 | 0.175*** | 0.888** |
|  | (0.045) | (0.038) | (0.406) |  | (0.045) | (0.038) | (0.431) |
| Depressive symptom (wave 2) | 0.002 | 0.001 | -0.005 |  | -0.001 | -0.000 | -0.024 |
|  | (0.004) | (0.005) | (0.034) |  | (0.004) | (0.005) | (0.041) |
| Dating experience (wave 2) | 0.145*** | 0.172*** | 1.389*** |  | 0.106** | 0.154*** | 1.158** |
|  | (0.042) | (0.043) | (0.377) |  | (0.050) | (0.052) | (0.451) |
| School fixed effects | yes | yes | yes |  | yes | yes | yes |
| First-stage F-statistic^3^ |  |  |  |  | 8.76 | 8.76 | 6.23 |
| Overidentifying restrictions |  |  |  |  | 0.552 | 0.872 | 0.495 |
| J-statistic (p-value) |  |  |  |  | (0.458) | (0.350) | (0.482) |
| Observations | 1,064 | 1,064 | 781 |  | 1,064 | 1,064 | 781 |
| R-squared | 0.085 | 0.101 | 0.146 |  |  |  |  |

**Notes**: ¹The instrument variables (IVs) in two-stage least squares model (2SLS) are pubertal timing that included two dummy variables for students’ pubertal timing being on-time and being early, respectively. The SEM exposure is a binary variable indicating that students ever exposed to SEM.

² The reference groups for these variables are: “below high school” for both *father's education* and *mother's education*; “below 30K” for *monthly income;* “ranked at 1-5” for *class rank in 7th grade*; “bad/very bad” for *health status*; Heteroskedasticity-robust standard errors in the parentheses clustered at the junior high school. *** p<0.01, ** p<0.05, * p<0.1.

^3^ The first-stage F-statistic is the F-statistic testing the hypothesis that the coefficients on the IVs (i.e., *pubertal timing*) equal zero in first stage of 2SLS. Overidentifying test with null hypothesis that the two instruments are consistent with each other is applied.

Table B Effects of Multi-modality SEM exposure on Risky Sexual Outcomes for Male

|  | OLS Results | | |  | 2SLS Results^1^ | | |
| --- | --- | --- | --- | --- | --- | --- | --- |
|  | Early | Unsafe | Number of |  | Early | Unsafe | Number of |
|  | sex debut | sex | sex partner |  | sex debut | sex | sex partner |
| Mean of dependent variable | 0.130 | 0.201 | 1.985 |  | 0.130 | 0.201 | 1.985 |
|  | (1) | (2) | (3) |  | (4) | (5) | (6) |
|  |  |  |  |  |  |  |  |
| Multi-modality SEM (wave 2) | 0.037*** | 0.035*** | 0.351*** |  | 0.099** | 0.069 | 0.764* |
|  | (0.009) | (0.011) | (0.068) |  | (0.045) | (0.064) | (0.416) |
| Father's education: high school^2^ | -0.004 | -0.022 | 0.123 |  | -0.014 | -0.027 | 0.028 |
|  | (0.025) | (0.034) | (0.243) |  | (0.027) | (0.033) | (0.282) |
| Father's education: junior college/above | 0.057 | 0.008 | -0.003 |  | 0.059 | 0.009 | -0.018 |
|  | (0.039) | (0.049) | (0.340) |  | (0.038) | (0.048) | (0.318) |
| Mom's education: high school^2^ | -0.001 | -0.014 | 0.055 |  | 0.009 | -0.008 | 0.072 |
|  | (0.029) | (0.040) | (0.264) |  | (0.032) | (0.040) | (0.253) |
| Mom's education: junior college/above | -0.006 | -0.027 | 0.160 |  | 0.006 | -0.020 | 0.200 |
|  | (0.049) | (0.036) | (0.350) |  | (0.050) | (0.038) | (0.330) |
| Monthly income: 30K-50K (NTD)^2^ | -0.012 | -0.050 | -0.289 |  | -0.010 | -0.049 | -0.271 |
|  | (0.044) | (0.041) | (0.261) |  | (0.043) | (0.040) | (0.270) |
| Monthly income: 50K-100K | -0.007 | -0.017 | -0.283 |  | -0.013 | -0.021 | -0.294 |
|  | (0.043) | (0.042) | (0.303) |  | (0.041) | (0.043) | (0.290) |
| Monthly income: 100K-150K | -0.007 | -0.028 | 0.254 |  | -0.017 | -0.034 | 0.217 |
|  | (0.047) | (0.059) | (0.618) |  | (0.045) | (0.060) | (0.554) |
| Monthly income: above 150K | 0.015 | 0.044 | 0.616 |  | -0.026 | 0.021 | 0.422 |
|  | (0.075) | (0.088) | (0.847) |  | (0.072) | (0.107) | (0.865) |
| Intact Family (wave 2) | 0.014 | 0.005 | 0.011 |  | 0.023 | 0.010 | 0.058 |
|  | (0.035) | (0.038) | (0.291) |  | (0.034) | (0.038) | (0.313) |
| Number of sibling (wave 1) | 0.007 | -0.022 | -0.240** |  | 0.006 | -0.022 | -0.228* |
|  | (0.017) | (0.016) | (0.118) |  | (0.017) | (0.015) | (0.117) |
| Only child (wave 1) | -0.010 | -0.017 | -0.458 |  | -0.003 | -0.012 | -0.428 |
|  | (0.047) | (0.071) | (0.483) |  | (0.045) | (0.066) | (0.447) |
| Presence of older sibling (wave 1) | 0.004 | 0.014 | -0.078 |  | -0.003 | 0.009 | -0.110 |
|  | (0.030) | (0.027) | (0.201) |  | (0.031) | (0.026) | (0.198) |
| Parental control (wave1) | -0.002 | -0.002 | 0.102* |  | -0.003 | -0.003 | 0.089 |
|  | (0.006) | (0.006) | (0.057) |  | (0.006) | (0.007) | (0.058) |
| Family cohesion (wave 1) | -0.002 | -0.004 | -0.030 |  | 0.001 | -0.003 | -0.020 |
|  | (0.003) | (0.004) | (0.027) |  | (0.003) | (0.005) | (0.027) |
| Class rank in 7th grade: 6-10 (wave 1)^2^ | 0.065* | 0.065 | 0.530* |  | 0.055 | 0.059 | 0.399 |
|  | (0.035) | (0.040) | (0.285) |  | (0.036) | (0.041) | (0.339) |
| Class rank in 7th grade: 11-20 | 0.063** | 0.080** | 0.495* |  | 0.056* | 0.075** | 0.402 |
|  | (0.031) | (0.034) | (0.268) |  | (0.032) | (0.034) | (0.313) |
| Class rank in 7th grade: over 20 | 0.128*** | 0.137*** | 0.751*** |  | 0.114*** | 0.129*** | 0.629** |
|  | (0.038) | (0.042) | (0.258) |  | (0.040) | (0.041) | (0.292) |
| Health status: fair (wave 2)^2^ | 0.048 | 0.121** | 0.470 |  | 0.033 | 0.112*** | 0.527 |
|  | (0.048) | (0.045) | (0.371) |  | (0.049) | (0.043) | (0.383) |
| Health status: good/very good | 0.070 | 0.175*** | 0.832** |  | 0.058 | 0.168*** | 0.882** |
|  | (0.043) | (0.040) | (0.397) |  | (0.043) | (0.042) | (0.420) |
| Depressive symptom (wave 2) | 0.001 | 0.001 | -0.003 |  | -0.002 | -0.000 | -0.014 |
|  | (0.004) | (0.005) | (0.033) |  | (0.004) | (0.005) | (0.035) |
| Dating experience (wave 2) | 0.129*** | 0.165*** | 1.402*** |  | 0.091* | 0.144** | 1.249*** |
|  | (0.043) | (0.043) | (0.372) |  | (0.051) | (0.056) | (0.402) |
| School fixed effects | yes | yes | yes |  | yes | yes | yes |
| First-stage F-statistic^3^ |  |  |  |  | 8.25 | 8.25 | 6.53 |
| Overidentifying restrictions |  |  |  |  | 0.502 | 0.722 | 0.473 |
| J-statistic (p-value) |  |  |  |  | (0.479) | (0.396) | (0.492) |
| Observations | 1,064 | 1,064 | 781 |  | 1,064 | 1,064 | 781 |
| R-squared | 0.106 | 0.106 | 0.156 |  |  |  |  |

**Notes**: ¹The instrument variables (IVs) in two-stage least squares model (2SLS) are pubertal timing that included two dummy variables for students’ pubertal timing being on-time and being early, respectively. The Multi-modality SEM exposure is the multiple type of SEM exposure (ranged from 0 to 6).

² The reference groups for these variables are: “below high school” for both *father's education* and *mother's education*; “below 30K” for *monthly income;* “ranked at 1-5” for *class rank in 7th grade*; “bad/very bad” for *health status*; Heteroskedasticity-robust standard errors in the parentheses clustered at the junior high school. *** p<0.01, ** p<0.05, * p<0.1.

^3^ The first-stage F-statistic is the F-statistic testing the hypothesis that the coefficients on the IVs (i.e., *pubertal timing*) equal zero in first stage of 2SLS. Overidentifying test with null hypothesis that the two instruments are consistent with each other is applied.

Table C Effects of SEM exposure on Risky Sexual Outcomes for Female

|  | OLS Results | | |  | 2SLS Results^1^ | | |
| --- | --- | --- | --- | --- | --- | --- | --- |
|  | Early | Unsafe | Number of |  | Early | Unsafe | Number of |
|  | sex debut | sex | sex partner |  | sex debut | sex | sex partner |
| Mean of dependent variable | 0.107 | 0.160 | 1.509 |  | 0.107 | 0.160 | 1.509 |
|  | (1) | (2) | (3) |  | (4) | (5) | (6) |
|  |  |  |  |  |  |  |  |
| SEM exposure (wave 2) | 0.050** | 0.083*** | 0.512** |  | 0.610* | 0.540 | 2.631 |
|  | (0.020) | (0.019) | (0.199) |  | (0.364) | (0.426) | (1.885) |
| Father's education: high school^2^ | 0.038 | 0.025 | 0.247 |  | 0.027 | 0.016 | 0.236 |
|  | (0.029) | (0.029) | (0.198) |  | (0.033) | (0.034) | (0.212) |
| Father's education: junior college/above | 0.019 | -0.039 | 0.095 |  | 0.039 | -0.022 | 0.264 |
|  | (0.032) | (0.039) | (0.279) |  | (0.040) | (0.047) | (0.357) |
| Mom's education: high school^2^ | -0.012 | 0.000 | 0.136 |  | -0.033 | -0.017 | 0.073 |
|  | (0.026) | (0.033) | (0.195) |  | (0.038) | (0.045) | (0.259) |
| Mom's education: junior college/above | 0.027 | 0.089** | 0.042 |  | 0.022 | 0.086* | 0.020 |
|  | (0.031) | (0.043) | (0.359) |  | (0.045) | (0.051) | (0.379) |
| Monthly income: 30K-50K (NTD)^2^ | 0.012 | 0.014 | 0.215 |  | -0.031 | -0.022 | -0.041 |
|  | (0.033) | (0.037) | (0.291) |  | (0.054) | (0.062) | (0.404) |
| Monthly income: 50K-100K | 0.021 | 0.012 | 0.540 |  | -0.012 | -0.015 | 0.301 |
|  | (0.034) | (0.038) | (0.327) |  | (0.048) | (0.051) | (0.409) |
| Monthly income: 100K-150K | -0.031 | 0.011 | 0.107 |  | -0.099 | -0.045 | -0.194 |
|  | (0.043) | (0.047) | (0.419) |  | (0.088) | (0.096) | (0.625) |
| Monthly income: above 150K | 0.058 | 0.046 | 0.621 |  | -0.019 | -0.017 | 0.267 |
|  | (0.083) | (0.085) | (0.783) |  | (0.117) | (0.117) | (0.878) |
| Intact Family (wave 2) | -0.144*** | -0.149*** | -1.068** |  | -0.045 | -0.068 | -0.812 |
|  | (0.049) | (0.050) | (0.439) |  | (0.084) | (0.093) | (0.517) |
| Number of sibling (wave 1) | 0.003 | -0.005 | -0.144 |  | 0.001 | -0.007 | -0.117 |
|  | (0.012) | (0.016) | (0.101) |  | (0.015) | (0.019) | (0.123) |
| Only child (wave 1) | -0.048 | -0.069 | -0.554 |  | -0.024 | -0.050 | -0.649 |
|  | (0.060) | (0.066) | (0.690) |  | (0.073) | (0.077) | (0.591) |
| Presence of older sibling (wave 1) | -0.017 | 0.026 | -0.140 |  | -0.005 | 0.035 | -0.089 |
|  | (0.018) | (0.021) | (0.205) |  | (0.026) | (0.025) | (0.224) |
| Parental control (wave1) | 0.003 | 0.003 | 0.051 |  | 0.001 | 0.002 | 0.031 |
|  | (0.005) | (0.007) | (0.067) |  | (0.008) | (0.008) | (0.064) |
| Family cohesion (wave 1) | -0.008** | -0.010*** | -0.057** |  | -0.001 | -0.004 | -0.028 |
|  | (0.003) | (0.003) | (0.026) |  | (0.006) | (0.007) | (0.041) |
| Class rank in 7th grade: 6-10 (wave 1)^2^ | 0.037 | -0.007 | 0.316 |  | 0.051 | 0.004 | 0.484 |
|  | (0.029) | (0.038) | (0.285) |  | (0.043) | (0.050) | (0.302) |
| Class rank in 7th grade: 11-20 | 0.015 | -0.007 | 0.515** |  | 0.031 | 0.006 | 0.599** |
|  | (0.028) | (0.031) | (0.242) |  | (0.042) | (0.046) | (0.264) |
| Class rank in 7th grade: over 20 | 0.053 | 0.037 | 0.440* |  | 0.066 | 0.048 | 0.568** |
|  | (0.032) | (0.036) | (0.251) |  | (0.043) | (0.042) | (0.277) |
| Health status: fair (wave 2)^2^ | 0.012 | 0.035 | 0.333 |  | 0.008 | 0.031 | 0.353 |
|  | (0.045) | (0.053) | (0.338) |  | (0.051) | (0.060) | (0.351) |
| Health status: good/very good | 0.042 | 0.064 | 0.469 |  | 0.027 | 0.052 | 0.418 |
|  | (0.052) | (0.054) | (0.367) |  | (0.066) | (0.065) | (0.391) |
| Depressive symptom (wave 2) | 0.003 | 0.005 | 0.051** |  | -0.004 | -0.001 | 0.028 |
|  | (0.004) | (0.004) | (0.021) |  | (0.007) | (0.006) | (0.030) |
| Dating experience (wave 2) | 0.048 | 0.161*** | 1.250*** |  | -0.021 | 0.105 | 1.059*** |
|  | (0.039) | (0.049) | (0.352) |  | (0.070) | (0.070) | (0.372) |
| School fixed effects | yes | yes | yes |  | yes | yes | yes |
| First-stage F-statistic^3^ |  |  |  |  | 2.63 | 2.63 | 3.03 |
| Overidentifying restrictions |  |  |  |  | 0.616 | 0.037 | 4.321 |
| J-statistic (p-value) |  |  |  |  | (0.433) | (0.847) | (0.034) |
| Observations | 990 | 990 | 696 |  | 990 | 990 | 696 |
| R-squared | 0.110 | 0.122 | 0.193 |  |  |  |  |

**Notes**: ¹The instrument variables (IVs) in two-stage least squares model (2SLS) are pubertal timing that included two dummy variables for students’ pubertal timing being on-time and being early, respectively. The SEM exposure is a binary variable indicating that students ever exposed to SEM.

² The reference groups for these variables are: “below high school” for both *father's education* and *mother's education*; “below 30K” for *monthly income;* “ranked at 1-5” for *class rank in 7th grade*; “bad/very bad” for *health status*; Heteroskedasticity-robust standard errors in the parentheses clustered at the junior high school. *** p<0.01, ** p<0.05, * p<0.1.

^3^ The first-stage F-statistic is the F-statistic testing the hypothesis that the coefficients on the IVs (i.e., *pubertal timing*) equal zero in first stage of 2SLS. Overidentifying test with null hypothesis that the two instruments are consistent with each other is applied.

Table D Effects of Multi-modality SEM exposure on Risky Sexual Outcomes for Female

|  | OLS Results | | |  | 2SLS Results^1^ | | |
| --- | --- | --- | --- | --- | --- | --- | --- |
|  | Early | Unsafe | Number of |  | Early | Unsafe | Number of |
|  | sex debut | sex | sex partner |  | sex debut | sex | sex partner |
| Mean of dependent variable | 0.107 | 0.160 | 1.509 |  | 0.107 | 0.160 | 1.509 |
|  | (1) | (2) | (3) |  | (4) | (5) | (6) |
|  |  |  |  |  |  |  |  |
| Multi-modality SEM (wave 2) | 0.024*** | 0.045*** | 0.379*** |  | 0.302* | 0.275 | 1.020 |
|  | (0.008) | (0.011) | (0.112) |  | (0.167) | (0.202) | (0.778) |
| Father's education: high school^2^ | 0.034 | 0.017 | 0.171 |  | -0.024 | -0.030 | 0.037 |
|  | (0.029) | (0.030) | (0.198) |  | (0.051) | (0.059) | (0.201) |
| Father's education: junior college/above | 0.016 | -0.044 | 0.070 |  | 0.005 | -0.053 | 0.099 |
|  | (0.032) | (0.037) | (0.266) |  | (0.043) | (0.039) | (0.271) |
| Mom's education: high school^2^ | -0.012 | -0.000 | 0.129 |  | -0.037 | -0.021 | 0.091 |
|  | (0.025) | (0.033) | (0.191) |  | (0.033) | (0.042) | (0.210) |
| Mom's education: junior college/above | 0.024 | 0.084** | 0.004 |  | -0.011 | 0.055 | -0.069 |
|  | (0.031) | (0.042) | (0.340) |  | (0.052) | (0.056) | (0.363) |
| Monthly income: 30K-50K (NTD)^2^ | 0.013 | 0.015 | 0.182 |  | -0.023 | -0.015 | 0.021 |
|  | (0.032) | (0.036) | (0.281) |  | (0.041) | (0.046) | (0.348) |
| Monthly income: 50K-100K | 0.024 | 0.016 | 0.534 |  | 0.017 | 0.010 | 0.426 |
|  | (0.034) | (0.037) | (0.321) |  | (0.039) | (0.033) | (0.342) |
| Monthly income: 100K-150K | -0.030 | 0.012 | 0.100 |  | -0.082 | -0.031 | -0.034 |
|  | (0.043) | (0.046) | (0.419) |  | (0.074) | (0.079) | (0.508) |
| Monthly income: above 150K | 0.057 | 0.042 | 0.559 |  | -0.035 | -0.034 | 0.309 |
|  | (0.083) | (0.082) | (0.787) |  | (0.127) | (0.120) | (0.844) |
| Intact Family (wave 2) | -0.146*** | -0.149*** | -1.050** |  | -0.055 | -0.074 | -0.916** |
|  | (0.050) | (0.051) | (0.425) |  | (0.079) | (0.080) | (0.466) |
| Number of sibling (wave 1) | 0.003 | -0.005 | -0.138 |  | 0.005 | -0.003 | -0.119 |
|  | (0.012) | (0.016) | (0.099) |  | (0.014) | (0.018) | (0.110) |
| Only child (wave 1) | -0.047 | -0.067 | -0.560 |  | -0.012 | -0.038 | -0.611 |
|  | (0.059) | (0.063) | (0.654) |  | (0.065) | (0.070) | (0.532) |
| Presence of older sibling (wave 1) | -0.018 | 0.023 | -0.158 |  | -0.021 | 0.021 | -0.168 |
|  | (0.018) | (0.021) | (0.207) |  | (0.030) | (0.030) | (0.216) |
| Parental control (wave1) | 0.003 | 0.003 | 0.056 |  | 0.003 | 0.003 | 0.055 |
|  | (0.005) | (0.007) | (0.065) |  | (0.008) | (0.008) | (0.061) |
| Family cohesion (wave 1) | -0.008** | -0.009*** | -0.048* |  | 0.003 | 0.000 | -0.019 |
|  | (0.003) | (0.003) | (0.025) |  | (0.006) | (0.009) | (0.046) |
| Class rank in 7th grade: 6-10 (wave 1)^2^ | 0.037 | -0.007 | 0.332 |  | 0.054 | 0.007 | 0.429 |
|  | (0.029) | (0.038) | (0.272) |  | (0.047) | (0.049) | (0.276) |
| Class rank in 7th grade: 11-20 | 0.016 | -0.004 | 0.560** |  | 0.047 | 0.021 | 0.672** |
|  | (0.027) | (0.031) | (0.248) |  | (0.045) | (0.050) | (0.269) |
| Class rank in 7th grade: over 20 | 0.053 | 0.038 | 0.494* |  | 0.070 | 0.052 | 0.637** |
|  | (0.033) | (0.037) | (0.251) |  | (0.046) | (0.044) | (0.281) |
| Health status: fair (wave 2)^2^ | 0.014 | 0.038 | 0.349 |  | 0.030 | 0.051 | 0.385 |
|  | (0.045) | (0.052) | (0.328) |  | (0.061) | (0.061) | (0.326) |
| Health status: good/very good | 0.043 | 0.065 | 0.455 |  | 0.033 | 0.056 | 0.411 |
|  | (0.051) | (0.053) | (0.358) |  | (0.068) | (0.063) | (0.354) |
| Depressive symptom (wave 2) | 0.003 | 0.004 | 0.044** |  | -0.009 | -0.005 | 0.023 |
|  | (0.004) | (0.004) | (0.021) |  | (0.009) | (0.010) | (0.034) |
| Dating experience (wave 2) | 0.048 | 0.160*** | 1.205*** |  | -0.023 | 0.102 | 1.049*** |
|  | (0.039) | (0.050) | (0.364) |  | (0.070) | (0.070) | (0.378) |
| School fixed effects | yes | yes | yes |  | yes | yes | yes |
| First-stage F-statistic^3^ |  |  |  |  | 2.00 | 2.00 | 3.35 |
| Overidentifying restrictions |  |  |  |  | 0.729 | 0.097 | 5.87 |
| J-statistic (p-value) |  |  |  |  | (0.393) | (0.755) | (0.015) |
| Observations | 990 | 990 | 696 |  | 990 | 990 | 696 |
| R-squared | 0.110 | 0.126 | 0.212 |  |  |  |  |

**Notes**: ¹The instrument variables (IVs) in two-stage least squares model (2SLS) are pubertal timing that included two dummy variables for students’ pubertal timing being on-time and being early, respectively. The Multi-modality SEM exposure is the multiple type of SEM exposure (ranged from 0 to 6).

² The reference groups for these variables are: “below high school” for both *father's education* and *mother's education*; “below 30K” for *monthly income;* “ranked at 1-5” for *class rank in 7th grade*; “bad/very bad” for *health status*; Heteroskedasticity-robust standard errors in the parentheses clustered at the junior high school. *** p<0.01, ** p<0.05, * p<0.1.

^3^ The first-stage F-statistic is the F-statistic testing the hypothesis that the coefficients on the IVs (i.e., *pubertal timing*) equal zero in first stage of 2SLS. Overidentifying test with null hypothesis that the two instruments are consistent with each other is applied.
